# EpCAM ectodomain EpEX is a ligand of EGFR that counteracts EGF-mediated epithelial-mesenchymal transition through modulation of phospho-ERK1/2 in head and neck cancers

**DOI:** 10.1371/journal.pbio.2006624

**Published:** 2018-09-27

**Authors:** Min Pan, Henrik Schinke, Elke Luxenburger, Gisela Kranz, Julius Shakhtour, Darko Libl, Yuanchi Huang, Aljaž Gaber, Miha Pavšič, Brigita Lenarčič, Julia Kitz, Mark Jakob, Sabina Schwenk-Zieger, Martin Canis, Julia Hess, Kristian Unger, Philipp Baumeister, Olivier Gires

**Affiliations:** 1 Department of Otorhinolaryngology, Head and Neck Surgery, Grosshadern Medical Center, Ludwig-Maximilians-University, Munich, Germany; 2 Department of Chemistry and Biochemistry, Faculty of Chemistry and Chemical Technology, University of Ljubljana, Ljubljana, Slovenia; 3 Department of Biochemistry, Molecular and Structural Biology, Institute Jožef Stefan, Ljubljana, Slovenia; 4 Institute of Pathology, University Medical Center, Göttingen, Germany; 5 Clinical Cooperation Group “Personalized Radiotherapy in Head and Neck Cancer“, Helmholtz Zentrum München, Research Center for Environmental Health (GmbH), Neuherberg, Germany; 6 Research Unit Radiation Cytogenetics, Helmholtz Zentrum München, Research Center for Environmental Health (GmbH), Neuherberg, Germany; Lincolns Inn Fields Laboratory, United Kingdom of Great Britain and Northern Ireland

## Abstract

Head and neck squamous cell carcinomas (HNSCCs) are characterized by outstanding molecular heterogeneity that results in severe therapy resistance and poor clinical outcome. Inter- and intratumoral heterogeneity in epithelial-mesenchymal transition (EMT) was recently revealed as a major parameter of poor clinical outcome. Here, we addressed the expression and function of the therapeutic target epidermal growth factor receptor (EGFR) and of the major determinant of epithelial differentiation epithelial cell adhesion molecule (EpCAM) in clinical samples and in vitro models of HNSCCs. We describe improved survival of EGFR^low^/EpCAM^high^ HNSCC patients (*n* = 180) and provide a molecular basis for the observed disparities in clinical outcome. EGF/EGFR have concentration-dependent dual capacities as inducers of proliferation and EMT through differential activation of the central molecular switch phosphorylated extracellular signal–regulated kinase 1/2 (pERK1/2) and EMT transcription factors (EMT-TFs) Snail, zinc finger E-box-binding homeobox 1 (Zeb1), and Slug. Furthermore, soluble ectodomain of EpCAM (EpEX) was identified as a ligand of EGFR that activates pERK1/2 and phosphorylated AKT (pAKT) and induces EGFR-dependent proliferation but represses EGF-mediated EMT, Snail, Zeb1, and Slug activation and cell migration. EMT repression by EpEX is realized through competitive modulation of pERK1/2 activation strength and inhibition of EMT-TFs, which is reflected in levels of pERK1/2 and its target Slug in clinical samples. Accordingly, high expression of pERK1/2 and/or Slug predicted poor outcome of HNSCCs. Hence, EpEX is a ligand of EGFR that induces proliferation but counteracts EMT mediated by the EGF/EGFR/pERK1/2 axis. Therefore, the emerging EGFR/EpCAM molecular cross talk represents a promising target to improve patient-tailored adjuvant treatment of HNSCCs.

## Author summary

Head and neck squamous cell carcinomas (HNSCCs) display poor survival, with death rates above 55%. Major factors affecting survival are metastases’ formation and therapy resistance. Phenotypic changes during partial epithelial-mesenchymal transition (EMT) provide tumor cells with increased migration, invasion, and therapy resistance. Understanding molecular mechanisms of EMT, as a central process of the metastatic cascade and the development of therapy resistance, is therefore important. In the present work, we identified molecular cross talk between epidermal growth factor receptor (EGFR) and epithelial cell adhesion molecule (EpCAM) as a novel determinant of clinical outcome in HNSCCs. Low levels of EGFR but high levels of EpCAM (EGFR^low^/EpCAM^high^) were associated with favorable prognosis, with survival rates above 90%, whereas EGFR^high^/EpCAM^low^ correlated with poor survival, below 10%. EGFR was shown to have a concentration-dependent capacity to induce proliferation and EMT. Proteolytic cleavage of the extracellular domain of EpCAM (EpEX) produces a ligand of EGFR that induces EGFR-dependent proliferation but counteracts EGF-induced EMT. We delineate an EGFR/extracellular signal–regulated kinase 1/2 (ERK1/2)/EpCAM signaling axis that may be a promising therapeutic target for HNSCCs.

## Introduction

Head and neck squamous cell carcinomas (HNSCCs) are the sixth-most-common carcinomas worldwide, with a poor 45% survival rate at 5 y [1], owing to early recurrence due to treatment failure and to the frequent presence of locoregional lymph node metastases at initial diagnosis [2]. Therefore, the requirement to control metastatic spread and to overcome therapy resistance represents major challenges and promises in HNSCC treatment. Furthermore, improvements of stratification options for patients beyond the current tumor, node, metastasis (TNM) classification and human papillomavirus (HPV) infection status are in high demand, in order to deliver patient-tailored precision medicine. In-depth knowledge of molecular processes that underlie metastasis formation and therapy resistance is thus of paramount importance to achieve best possible adjustment of adjuvant and palliative treatment of HNSCC patients who experience recurrences and metastases and, eventually, to improve clinical outcome. A mechanism central to metastatic spread and treatment resistance is referred to as epithelial-mesenchymal transition (EMT), which is a differentiation program that allows cells to acquire the migratory capacity initially described in embryonic development [3]. Cancer cells undergo a partial phenotypic EMT shift to progeny with enhanced mesenchymal features, i.e., gain of migratory and invasive potential, improved resistance to radiation and chemotherapeutic drugs, and development of cancer stem cell (CSC) features [4–6]. Accordingly, the use of multigene transcriptomic EMT signatures as a scoring system was demonstrated to have predictive value in a variety of carcinomas [7].

HNSCCs are characterized by strong genetic heterogeneity [8], with an average of 130 mutations per tumor [9,10]. High intratumoral heterogeneity correlates with decreased overall survival (OS) [11] and provides cellular diversity, which fosters the emergence of cellular subpopulations equipped with enhanced EMT traits and treatment resistance [12–15]. Specifically, HNSCCs are composed of heterogeneous tumor cells with individual RNA transcript signatures related to cell cycle, stress response, hypoxia, epithelial differentiation, and partial EMT (pEMT) [16]. Malignant pEMT cells were preferentially located at leading edges of tumor areas, and their signature was reciprocal to that of the epithelial differentiation, which was codefined by high expression of the pan-carcinoma epithelial cell adhesion molecule (EpCAM) [16]. The identified pEMT program correlated with metastases formation and poor prognosis and was suggested to result from interactions of tumor cells with cancer-associated fibroblasts of the tumor microenvironment [16].

Despite such highly encouraging innovative data on molecular features of HNSCCs, therapeutic options to cope with metastasized and progressing HNSCCs at the molecular target level remain unsatisfying. Currently, epidermal growth factor receptor (EGFR) is the major target for therapeutic antibodies and inhibitor-based palliative treatment regimens aiming at controlling recurrent and metastatic disease in HNSCC patients. EGFR is frequently and strongly expressed in HNSCCs [9], has prognostic and predictive value [17], and serves as an anchor for Food and Drug Administration (FDA)-approved therapeutic antibodies and inhibitors targeting EGFR, including monoclonal antibody Cetuximab, which is primarily implemented into palliative treatment of metastatic head and neck, colon, and non-small-cell lung cancer patients [18–20]. However, clinical benefits deployed by inhibition of EGFR in HNSCCs remain insufficient because of highly advanced stages of disease, the development of multiple resistances [21], and pleiotropic cellular functions of EGFR in proliferation and cell differentiation, including the regulation of EMT [22–27]. Therefore, deepening the understanding of EGFR signaling towards proliferation and EMT in HNSCCs is advisable to improve treatment regimens.

In the present study, we describe a strong impact of differential EGFR and EpCAM expression on the clinical outcome of HNSCC patients. Low expression of EGFR combined with high expression of EpCAM correlated with substantially improved survival. We further defined a molecular basis for this disparity in outcome, in which the soluble extracellular domain of EpCAM (EpEX) acts as a ligand for EGFR, which induces signaling to extracellular signal–regulated kinase 1/2 (ERK1/2) and AKT. Unlike EGF, which has a concentration-dependent dual function in HNSCCs as an inducer of proliferation or EMT, EpEX induces EGFR-dependent proliferation but represses EGF-mediated EMT through reduction of ERK1/2 phosphorylation and of EMT transcription factors (EMT-TFs) Snail, zinc finger E-box-binding homeobox 1 (ZEB1), and Slug expression. Accordingly, phosphorylation of ERK1/2 and/or expression of Slug further defined HNSCC patients with dismal outcome. Hence, we disclose a regulatory signaling cross talk between EGFR and EpCAM in cancer cells that impacts proliferation and EMT and may thus have substantial repercussions on disease progression and outcome. As such, the EGFR/EpCAM axis represents a novel and promising candidate to further develop patient-tailored therapeutic approaches.

## Results

### EGFR and EpCAM define subpopulations of HNSCCs with differing clinical outcome

**Fig 1:**
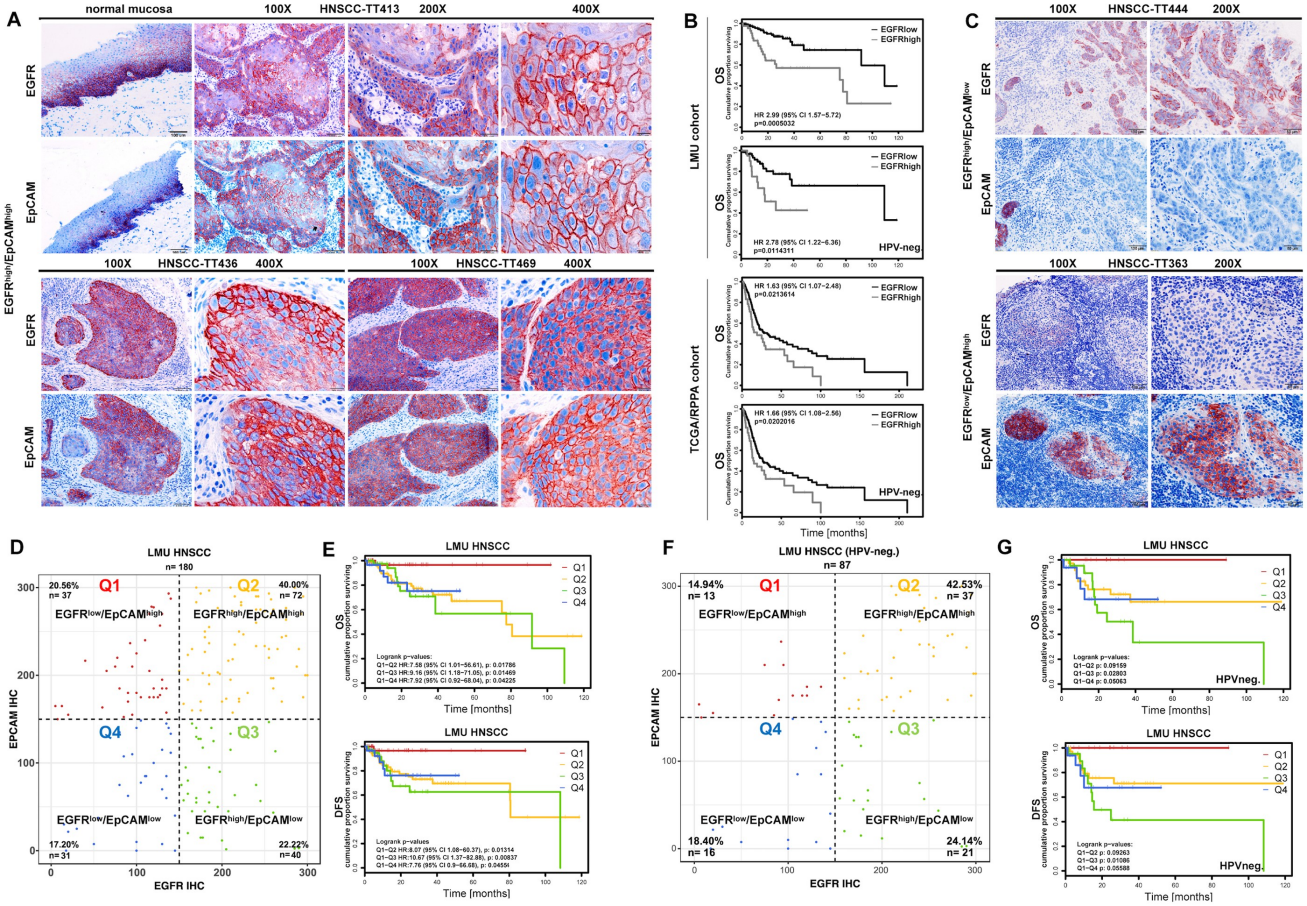
EGFR and EpCAM expression predicts differential clinical outcome of HNSCCs. (A, C) Expression of EGFR and EpCAM was assessed in serial cryosections of normal mucosa and primary HNSCCs by IHC staining. Shown are representative examples of EGFR^high^/EpCAM^high^ (A), EGFR^high^/EpCAM^low^, and EGFR^low^/EpCAM^high^ (C) tumors at 100×, 200×, and 400× magnifications. (B) OS probability of HNSCC patients from the LMU cohort (*n* = 180) and from a subcohort of the HNSCC TCGA cohort (*n* = 279) [9] was stratified according to EGFR expression (cutoff threshold at the third quartile). IHC scores were generated for the LMU cohort as described [28]. Protein expression data for EGFR from the RPPA data were used for the TCGA cohort. OS is represented as Kaplan-Meier survival curves with *p*-value, HR, and CI for the entire cohorts and the HPV-negative subcohorts. Supporting data are compiled in S1 Data. (D, F) IHC scores of EGFR and EpCAM expression were assessed in *n* = 180 primary HNSCCs of the LMU cohort (D) and in *n* = 87 HPV-negative primary HNSCCs of the LMU cohort (F). Expression correlation of EGFR and EpCAM is plotted and subdivided according to a cutoff threshold of 150 (score range 0–300). Numbers and percentages of patients within subgroups are indicated in each quadrant. (E, G) OS and DFS were stratified according to all four quadrants defined in D and F and are represented as Kaplan-Meier survival curves with *p*-values, HRs, and CIs. Supporting data are compiled in S1 Data. CI, confidence interval; DFS, disease-free survival; EGFR, epidermal growth factor receptor; EpCAM, epithelial cell adhesion molecule; HNSCC, head and neck squamous cell carcinoma; HPV, human papillomavirus; HR, hazard ratio; IHC, immunohistochemistry; LMU, Ludwig-Maximilians-University; OS, overall survival; RPPA, reversed-phase protein atlas; TCGA, the Cancer Genome Atlas.

EGFR and EpCAM expression was assessed in serial cryosections of primary HNSCCs within a cohort of the head and neck department at the Ludwig-Maximilians-University (LMU) of Munich, Germany (LMU cohort; *n* = 180; S1 Table). EGFR and EpCAM were expressed in suprabasal layers of normal mucosa and were strongly expressed in primary carcinomas, with a frequent coexpression at the single-cell level (Fig 1A). Expression levels of EGFR and EpCAM were assessed with an immunohistochemistry (IHC) scoring (IHC score 0–300) [28], and EGFR levels served to stratify patients. With the third quartile as the cutoff threshold, EGFR^high^ defined patients with reduced OS across all patients (*n* = 180) (Fig 1B). Chronic infection with high-risk HPV predicts improved outcome of HNSCC patients [29] and is therefore an accepted clinical factor that has recently been implemented in the American Joint Committee on Cancer (AJCC) 8th Edition of the TNM classification of patients [30,31]. In order to determine the predictive capacity of EGFR independently of HPV status as a potential confounder, OS was calculated for the HPV-negative LMU subcohort (*n* = 87). Similar to the entire cohort, EGFR^high^ predicted poor survival of HNSCCs in HPV-negative cases (Fig 1B). Stratification of patients from the Cancer Genome Atlas (TCGA) HNSCC cohort [9], using reversed-phase protein atlas data on EGFR expression, confirmed similar clinical outcome and poor OS of EGFR^high^ patients, comparable with the LMU cohort (Fig 1B).

EpCAM has been described as a sensitive marker for an epithelial differentiation signature in HNSCCs [16], which prompted us to analyze its expression in relation to EGFR. Besides tumors with a concurrent strong expression of both antigens, HNSCCs with reciprocal expression patterns of EGFR and EpCAM were identified as EGFR^high^/EpCAM^low^ and EGFR^low^/EpCAM^high^ (Fig 1C). Subgroups of patients with EGFR^high^/EpCAM^low^, EGFR^low^/EpCAM^high^, EGFR^high^/EpCAM^high^, and EGFR^low^/EpCAM^low^ expression patterns were classified based on IHC scoring using a cutoff threshold of 150. EGFR^high^/EpCAM^high^ (*n* = 72) represented 40.00% of primary tumors, EGFR^low^/EpCAM^low^ (*n* = 31) 17.20%, and differential EGFR^low^/EpCAM^high^ (*n* = 37) and EGFR^high^/EpCAM^low^ (*n* = 40) expression 20.56% and 22.22%, respectively (Fig 1D). OS and disease-free survival (DFS; median follow-up 23 mo) were analyzed in EGFR/EpCAM subgroups of patients. EGFR^low^/EpCAM^high^ patients displayed significantly improved OS and DFS, as compared to all other subgroups (Fig 1E). Comparison of the two opposite subgroups of EGFR^low^/EpCAM^high^ and EGFR^high^/EpCAM^low^ patients disclosed OS above 90% and below 10%, respectively.

EGFR/EpCAM subgroups of patients were tested for associations with the clinical parameters tumor localization, grading, T stage, N stage, age, and p16 status, which is a surrogate marker for HPV infection [32]. Although statistically not significant (*p* = 0.055), HPV-positive patients, which associates with improved prognosis [33–35], were slightly enriched within the subgroup of EGFR^low^/EpCAM^high^ tumors (S1A Fig). Therefore, OS and DFS (median follow-up 19 mo) were analyzed in HPV-negative patients within the LMU cohort by censoring p16-positive and not-determined cases (*n* = 87). Repartition of HPV-negative patients into all four EGFR/EpCAM quadrants was similar to the full cohort (Fig 1F) and confirmed a significantly improved OS and DFS of EGFR^low^/EpCAM^high^ over EGFR^high^/EpCAM^low^ (Fig 1G). Additionally, the subgroup of EGFR^high^/EpCAM^low^ tumors was comprised of significantly more HNSCCs of the oral cavity, whereas EGFR^low^/EpCAM^high^ tumors comprised significantly fewer HNSCCs of the oral cavity (S1B Fig). In order to exclude a confounding impact of sublocalizations of tumors on the clinical outcome of the four EGFR/EpCAM patient subgroups, we analyzed the most prominent entity, i.e., oropharyngeal carcinomas (*n* = 105). Quadrant repartition was comparable to the full LMU cohort (S1C Fig). In confirmation of all results so far, EGFR^low^/EpCAM^high^ patients with oropharyngeal HNSCCs displayed considerably and significantly improved OS and DFS compared to EGFR^high^/EpCAM^low^ patients (S1D Fig). It must be noted that these differences in outcome remain present even in the case of oropharyngeal HNSCCs with a generally better prognosis. Hence, low expression of EGFR and high expression of EpCAM are markers of improved clinical outcome in HNSCC patients.

Induction of EGFR signals through EGF and further ligands results in the activation of major pathways rat sarcoma gene (Ras)/rapidly accelerated fibrosarcoma kinase (Raf)/MAPK–ERK kinase (MEK)/ERK and phosphoinositide-3 (PI3) kinase/AKT. Single and combinations of these pathways differentially promote proliferation, anti-apoptotic features, induction of EMT, and, as reported recently, activation of EpCAM regulated intramembrane proteolysis (RIP) [17,24,36,37]. EGF-mediated RIP of EpCAM was reported to lead to the release of the intracellular domain of EpCAM (EpICD) that, aside from fostering proliferation [38,39], induces an EMT program in endometrial carcinoma cells [36]. This prompted us to investigate potential functional interactions of EGFR and EpCAM that could provide a molecular rationale for the observed disparity in clinical outcome, including the improved survival of HPV-associated HNSCCs.

### EGF induces EMT in HNSCC cell lines, but induction of EpCAM RIP by EGF is not a common mechanism in carcinoma cells

**Fig 2:**
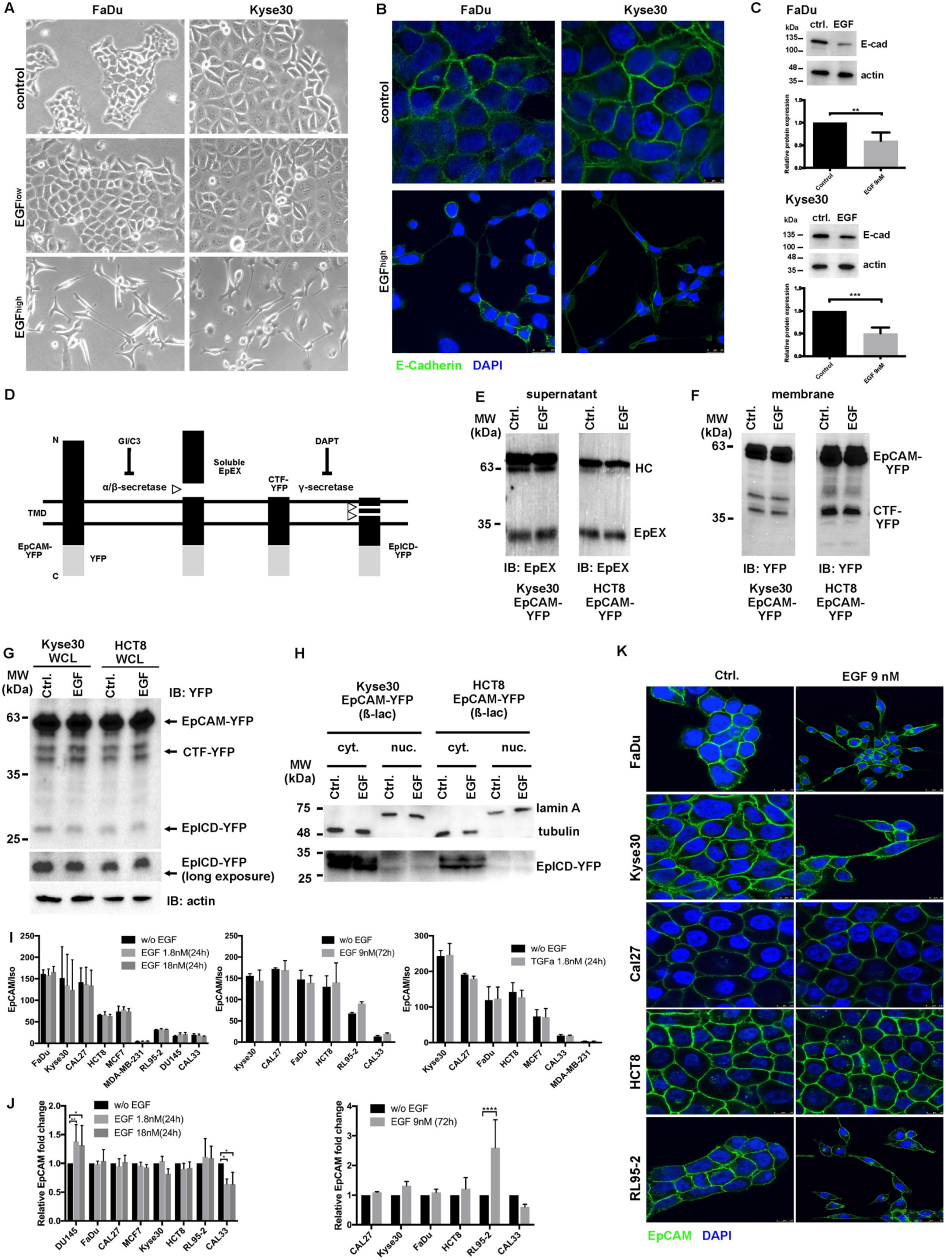
EGF promotes EMT but does not induce RIP of EpCAM. (A) FaDu and Kyse30 cells were treated with control media and a low (1.8 nM) and high (9 nM) dose of EGF. Cell morphology was assessed after 72 hr. Shown are representative micrographs at 100× magnification from 3 independent experiments. (B) FaDu and Kyse30 cells were treated with control media and a high (9 nM) dose of EGF. Expression of E-cadherin was assessed by immunofluorescence staining and confocal laser scanning microscopy after 72 hr. Shown are representative staining from 3 independent experiments. (C) FaDu and Kyse30 cells were treated with control media (“ctrl.”) and high (9 nM) dose of EGF. Expression of E-cadherin was assessed by IB after 72 hr. Shown are representative immunoblots (upper panels) and mean values with SDs from *n* = 3 independent experiments. ** *p*-value < 0.01, *** *p*-value < 0.001; paired Student *t* test. Supporting data are compiled in S1 Data. (D) Schematic representation of EpCAM RIP including proteases involved and inhibitors. (E) Immunoprecipitation of the EpEX from supernatants of Kyse30 and HCT8 cells expressing EpCAM-YFP with or without EGF 9 nM. Shown are representative results from *n* = 3 independent experiments. (F) Visualization of CTF-EpCAM-YFP in membrane isolates of Kyse30 and HCT8 cells expressing EpCAM-YFP with or without EGF 9 nM. Shown are representative results from *n* = 3 independent experiments. (G) Visualization of EpCAM-YFP, CTF-YFP, and EpICD-YFP in Kyse30 and HCT8 and Kyse30 cells expressing EpCAM-YFP with or without EGF 9 nM in the presence of proteasome inhibitor ß-lactone (“ß-lac,” 50 μM). Shown are representative results from *n* = 3 independent experiments. (H) Visualization of EpICD-YFP in cytoplasmic (“cyt.”) and nuclear fractions (“nuc.”) of Kyse30 and HCT8 cells expressing EpCAM-YFP with or without EGF 9 nM in the presence of proteasome inhibitor (“ß-lac,” 50 μM). Tubulin and lamin A staining served to control fractionated samples. Shown are representative results from *n* = 3 independent experiments. (I, J) Indicated cell lines were treated with EGF 1.8 nM and 18 nM for 24 hr, 9 nM for 72 hr, or 1.8 nM TGFα for 24 hr. Expression of EpCAM in control (“w/o EGF”) and treated cells was assessed by flow cytometry (I) and IB (J). Shown are ratios of EpCAM mean fluorescence intensities divided by control intensities (I; “EpCAM/iso”) and relative fold changes of EpCAM expression in whole-cell lysates (J; relative EpCAM fold change), where expression levels in control cells were set to 1 for comparison. Shown are means with SDs from *n* = 3 independent experiments. ** *p*-value < 0.01; paired Student *t* test. Supporting data are compiled in S1 Data. (K) Indicated cell lines were treated with EGF 9 nM for 72 hr, and cell surface expression of EpCAM was assessed by fluorescence immunostaining and laser scanning confocal microscopy. EpCAM: green, nuclei: blue (DAPI). Shown are representative results from *n* = 3 independent experiments with multiple areas analyzed. CTF, C-terminal fragment; DAPT, N-[N-(3,5-Difluorophenacetyl)-L-alanyl]-S-phenylglycine t-butyl ester; EGF, epidermal growth factor; EMT, epithelial-mesenchymal transition; EpCAM, epithelial cell adhesion molecule; EpEX, extracellular domain of EpCAM; IB, immunoblotting; MW, molecular mass; RIP, regulated intramembrane proteolysis; SD, standard deviation; TGFα, transforming growth factor alpha; YFP, yellow fluorescent protein.

In order to address whether EGF/EGFR signaling induces EMT in HNSCC cells, FaDu and Kyse30 cells were treated with low and high concentrations of EGF (1.8 nM and 9 nM). EGF^low^ (1.8 nM) corresponded to concentrations reportedly inducing EMT in endometrial carcinoma cells [36]. No signs of morphological changes along the EMT were observed after 72 hr of treatment with EGF^low^; however, EGF^high^ (9 nM) induced strong loss of cell–cell contact and the adoption of a spindle-shape morphology in FaDu and Kyse30 cells (Fig 2A). Immunofluorescence staining and confocal imaging of the major cell adhesion molecule and EMT target E-cadherin confirmed a shift to a mesenchymal appearance and partial loss of E-cadherin expression in both cell lines (Fig 2B). Reduction of E-cadherin expression, which is considered a hallmark of an EMT shift [3,40,41], was also observed in whole-cell extracts of EGF^high^-treated FaDu and Kyse30 cells (Fig 2C). Hence, EGF induces morphological changes reminiscent of EMT and loss of E-cadherin in HNSCC cell lines.

EGF treatment of endometrial carcinoma cells was reported to induce an EGFR-dependent RIP of EpCAM, resulting in complete EpEX shedding from the membrane and in release of EpICD, which serves as a nuclear transcriptional inducer of an EMT program [36]. EpICD was furthermore described as a regulator of proliferation and stem cell differentiation [38,39,42,43]. Therefore, we aimed at evaluating a potential contribution of EGFR-mediated RIP of EpCAM to the differential clinical outcome defined in Fig 1 and S1 Fig. EGFR and EpCAM were highly coexpressed at the cell surface of HNSCC/esophageal lines (FaDu, Cal27, Kyse30) and colon cell line HCT8 (S2A Fig). Membranous colocalization of EGFR and EpCAM was confirmed with dual immunofluorescence staining of FaDu and Cal27 cells (S2B Fig).

First, we analyzed the potential of EGF to induce RIP of EpCAM at the level of ectodomain shedding (EpEX), C-terminal fragment (CTF), and EpICD generation (see scheme in Fig 2D). Kyse30 and HCT8 cells stably expressing a human fusion of EpCAM with yellow fluorescence protein (EpCAM-YFP) were untreated or treated with EGF (9 nM, 72 hr). Treatment of Kyse30 and HCT8 with EGF did promote neither EpEX shedding in cell supernatants (Fig 2E) nor formation of a membrane-associated CTF-EpCAM-YFP (Fig 2F). EGF treatment also did not foster the formation of EpICD in whole-cell lysates or in cytoplasmic and nuclear extracts of Kyse30 and HCT8 cells (Fig 2G and 2H), so we conclude that EGFR-dependent RIP of EpCAM is not a common process in carcinoma cells. In order to certify that concentrations used by Hsu and colleagues would not induce RIP of EpCAM, despite no effects on EMT in the cell lines studied here, we repeated treatment of HCT8 cells with 1.8 nM EGF for 24 hr. Similarly, we could not observe any increase in ectodomain shedding (S3A Fig), CTF formation (S3B Fig), and EpICD release (S3C Fig).

To further test effects of EGF on EpCAM expression/RIP in a broader panel of cell lines, cell surface expression of EpCAM was assessed in HNSCCs (FaDu, Kyse30, Cal27, Cal33) and in colon (HCT8), breast (MCF7, MDA-MB-231), endometrial (RL95-2), and prostate (Du145) cancer cells following EGF treatment. Independently of initial expression levels of EGFR and EpCAM, quantification of EGF effects on cell surface expression of EpCAM did not disclose any significant difference (Fig 2I, S4A–S4C Fig). Treatment of Kyse30, Cal27, Cal33, FaDu, HCT8, and RL95-2 cells with 1.8 or 18 nM EGF for 24 hr or a second ligand of EGFR transforming growth factor alpha (TGFα) (1.8 nM, 24 hr) did not reduce EpCAM cell surface expression (Fig 2I, 2J, S4B, S4C Fig). Treatment of cells with TGFα was conducted to ensure that the lack of EpCAM reduction following activation of EGFR through EGF was not due to requirements for different EGFR ligands in HNSCC cell lines. Treatment with a 10-fold-higher concentration of EGF (i.e., 18 nM), as compared to Hsu and colleagues [36], was used in order to rule out dosage effects in HNSCCs as compared to endometrial carcinoma cells. Furthermore, prolonged treatment with 9 nM for 72 hr did not affect EpCAM expression in the cell lines tested, with the exception of a slight induction of cell surface expression of EpCAM in RL95-2 cells (Fig 2I, right panel). Effects of 1.8 nM and 18 nM EGF treatment for 24 hr and 9 nM for 72 hr on EpCAM protein expression were analyzed in lysates of FaDu, Cal27, Cal33, Kyse30, MCF7, HCT8, Du145, and RL95-2 cells. Quantification of immunoblot results did reveal an induction of EpCAM expression levels in Du145 cells treated with 1.8 and 18 nM EGF and RL95-2 endometrial carcinoma cells with 9 nM EGF for 72 hr, and a slight reduction of EpCAM expression following 1.8 and 18 nM EGF treatment of Cal33 (Fig 2J, S4D and S4E Fig). Visualization of EpCAM at the cell surface of FaDu, Cal27, Kyse30, RL95-2, and HCT8 carcinoma cells using immunofluorescence staining with EpEX-specific antibodies and confocal laser scanning microscopy confirmed a retention of EpCAM at the cell surface after treatment with EGF 9 nM for 72 hr (Fig 2K) or with EGF 1.8 nM or 18 nM for 24 hr (S3D Fig).

Finally, in order to assess the impact of EGFR on EMT through regulation of EpCAM, we chose Kyse30 cells, which showed the highest EGFR and EpCAM expression and strong EMT phenotype following EGF^high^ treatment. Wild-type Kyse30, a stable transfectant expressing EpCAM-YFP, two different short hairpin RNA (shRNA) EpCAM knockdown clones, and an shRNA control clone were treated with EGF (1.8 and 9 nM). Initial expression levels of EpCAM and EpCAM-YFP were assessed by immunoblotting and confirmed a lack of EpCAM in knockdown clones and exogeneous expression of EpCAM-YFP (S5A Fig). Wild-type Kyse30 cells displayed concentration-dependent EMT, with morphological changes towards spindle-shaped cells lacking cell–cell contact after EGF treatment (S5B Fig). Overexpression of EpCAM-YFP reduced the extent of EMT, whereas knockdown of EpCAM rather promoted a minimal increase in EMT changes observed after EGF treatment and in controls after 48 hr (S5B Fig). Hence, EpCAM is dispensable for EGF-induced EMT, and consequently, RIP of EpCAM to generate EpICD, as an inducer of EMT-specific genes, does not appear as a major determinant of EMT induction by EGF.

### EpEX is an EGFR ligand that induces ERK1/2 and AKT phosphorylation

**Fig 3:**
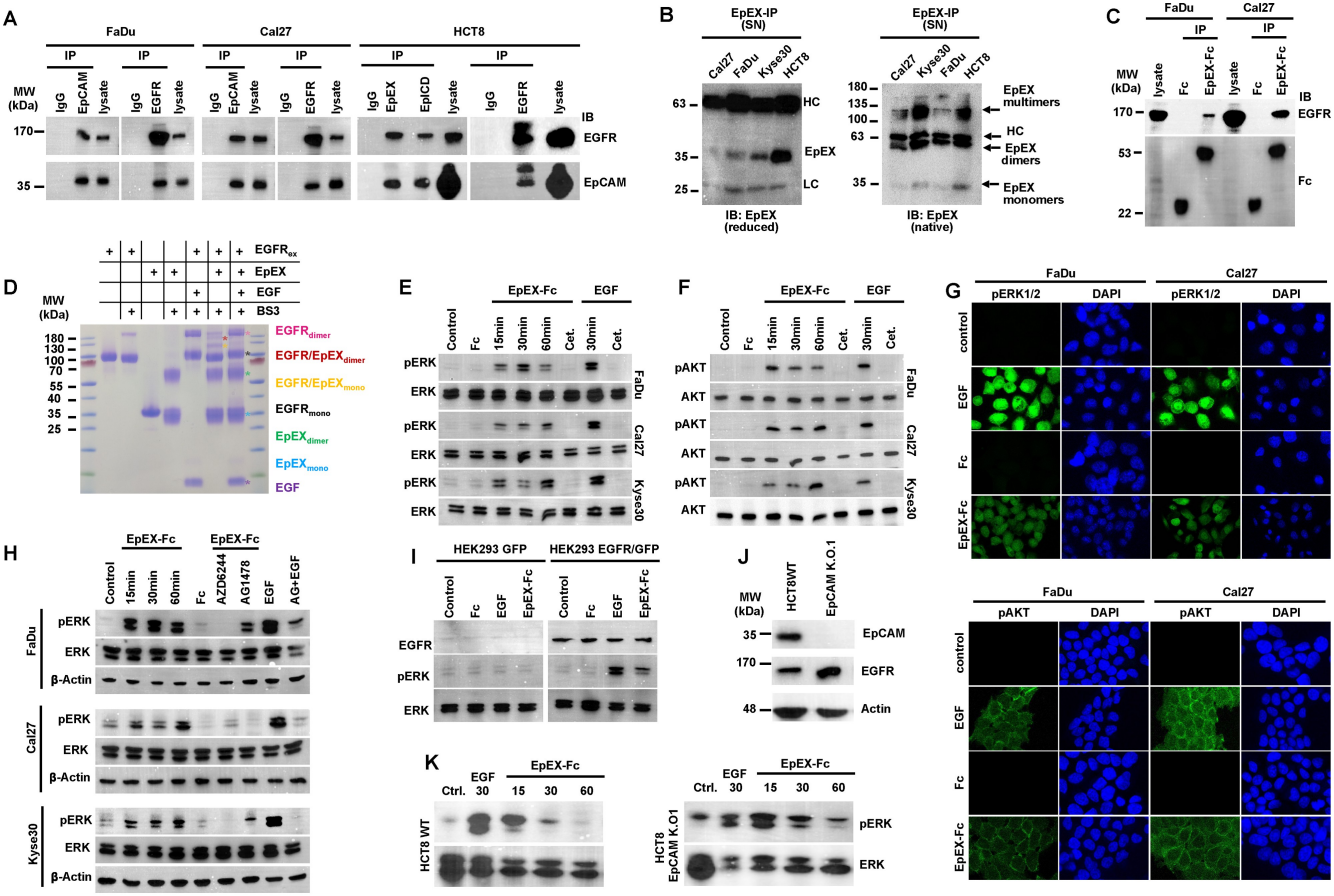
Soluble EpEX-Fc binds to EGFR and induces ERK1/2 and AKT. (A) Bidirectional co-immunoprecipitation (“IP”) of EGFR and EpCAM in whole-cell lysates of FaDu, Cal27, and HCT8 cells using EGFR- and EpCAM-specific antibodies. Isotype control antibody (“IgG”) served as control. Coimmunoprecipitated EGFR and EpCAM were visualized in immunoblotting with specific antibodies (“IB”), with whole-cell lysates as control (“lysate”). Shown are representative results from *n* = 3 independent experiments. (B) SNs of Cal27, Kyse30, FaDu, and HCT8 cells were immunoprecipitated with EpEX-specific antibodies and separated under reducing (left) and nonreducing native conditions (right), and EpEX was detected with specific antibodies. Antibody HCs and EpEX mono-, di-, and oligomers are indicated. Shown are representative results from *n* = 3 independent experiments. (C) EpEX-Fc or Fc were incubated with whole-cell lysates of FaDu and Cal27 and immobilized on protein A agarose beads, and protein complexes were separated on SDS-PAGE. Immunoprecipitated proteins were detected by immunoblotting (“IB”) with Fc- and EGFR-specific antibodies. Shown are representative results from *n* = 3 independent experiments. (D) EGFR_ex_ and EpEX were incubated in the presence or absence of cross-linker (BS3). Where indicated, EGF was added. Monomers, dimers, and EGFR_ex_/EpEX complexes are marked. Shown are representative results from *n* = 3 independent experiments. (E, F) FaDu, Cal27, and Kyse30 cells were kept untreated (control) or were treated with EpEX-Fc, Fc (10 nM), or EGF (1.8 nM) for the indicated time points. Where indicated, cells were additionally treated with Cetuximab (“Cet.”). Phosphorylation of ERK1/2 (E) and AKT (F) was assessed by immunoblotting with specific antibodies. Levels of ERK1/2 and AKT were assessed in parallel. Shown are representative results from *n* = 3 independent experiments. (G) FaDu and Cal27 cells were kept untreated or were treated with EGF (9 nM), Fc, or EpEX-Fc (10 nM) for 30 min, and phosphorylation of ERK1/2 and AKT was detected by immunofluorescence laser scanning confocal microscopy (ERK1/2 or AKT: green, nuclei: blue [DAPI]). Shown are representative results from *n* = 3 independent experiments. (H) FaDu, Cal27, and Kyse30 cells were kept untreated (control) or were treated with EpEX-Fc, Fc (10 nM), or EGF (1.8 nM) for the indicated time points (“EGF 30 min”). Where indicated, MEK1 inhibitor AZD6244 or EGFR inhibitor AG1478 were added. Levels of ERK1/2 and AKT were assessed in parallel. Shown are representative results from *n* = 3 independent experiments. (I) HEK293 cells were transiently transfected with GFP or EGFR expression plasmids and were either kept untreated (control) or were treated with EpEX-Fc, Fc (10 nM), or EGF (1.8 nM) for 30 min. Expression of EGFR and activation of ERK1/2 were assessed by immunoblotting. Levels of ERK1/2 were assessed in parallel. Shown are representative results from *n* = 3 independent experiments. (J) Expression of EpCAM and EGFR was assessed in HCT8WT and CRISPR-Cas9 EpCAM K.O.1 [48] by immunoblotting. Levels of actin were assessed in parallel. Shown are representative results from *n* = 3 independent experiments. (K) HCT8WT and CRISPR-Cas9 EpCAM K.O.1 cells were either kept untreated (control) or were treated with EpEX-Fc, Fc (10 nM), or EGF (1.8 nM) for 30 min. Activation of ERK1/2 was assessed by immunoblotting. Levels of ERK1/2 were assessed in parallel. Shown are representative results from *n* = 3 independent experiments. BS3, bisulfosuccinimidyl suberate; CRISPR-Cas9, clustered regularly interspaced short palindromic repeat/CRISPR-associated 9; EGF, epidermal growth factor; EGFR, EGF receptor; EGFR_ex_, extracellular domain of EGFR; EpCAM, epithelial cell adhesion molecule; EpCAM K.O.1, *EPCAM*-knockout clone 1; EpEX, extracellular domain of EpCAM; EpICD, intracellular domain of EpCAM; ERK1/2, extracellular signal–regulated kinase 1/2; Fc, fragment crystallizable region; GFP, green fluorescent protein; HC, heavy chain; HCT8WT, HCT8 wild type; HEK293, human embryonic kidney 293; IgG, immunoglobulin G; MW, molecular mass; pAKT, phosphorylated AKT; pERK, phosphorylated ERK; SN, supernatant.

To further address potential interactions of EGFR and EpCAM, bidirectional coimmunoprecipitation of endogenous proteins was performed. Precipitation of EpCAM in FaDu, Cal27, and HCT8 cells allowed for the coprecipitation of EGFR and vice versa (Fig 3A).

As was repeatedly reported, RIP of EpCAM is a process that occurs in cancer cells [38,39,44,45] and results in the presence of EpEX in the serum of cancer patients [46]. Immunoprecipitation of supernatants of Cal27, FaDu, Kyse30, and HCT8 cells with antibodies targeting EpEX confirmed the presence of EpEX (Fig 3B, left panel). In its native form, the EpEX forms heart-shaped homodimers [47], which was also observed for recombinant EpEX and soluble EpEX in cell culture supernatants of HCT8 cells [46]. Separation of immunoprecipitated EpEX from Cal27, FaDu, Kyse30, and HCT8 supernatants under nonreducing, native conditions confirmed the presence of EpEX mono-, di-, and oligomers (Fig 3B, right panel). To test a potential binding of EpEX to EGFR, EpEX was fused to the constant region of human immunoglobulin 1 as described [48], to generate EpEX-Fc (S6A Fig). EpEX-Fc was expressed in human embryonic kidney 293 (HEK293) cells and enriched from supernatants with high purity (S6B Fig). Specificity, the oligomeric state and N-glycosylation of recombinant EpEX-Fc were confirmed (S6C–S6E Fig). Oligomerization through the fragment crystallizable region (Fc) mimics the dimeric/oligomeric state of EpEX, with mono-, di-, and oligomers as observed in cell culture supernatants (See Fig 3B and [46]). EpEX-Fc and Fc served as baits to isolate interacting proteins from FaDu and Cal27 cell lysates. EpEX-Fc, but not Fc, interacted with full-length EGFR, suggesting that EpEX-Fc can act as a ligand for EGFR (Fig 3C). To further determine whether EpEX directly binds to the extracellular domain of EGFR (EGFR_ex_), cross-linking experiments were performed with purified recombinant EGFR_ex_ and EpEX. Cross-linking of EGFR_ex_ resulted in the generation of EGFR_ex_ dimers, which was further increased through addition of EGF (Fig 3D). Cross-linking of EpEX induced dimerization as described [47]. Incubation of EGFR_ex_ and EpEX induced the formation of a protein complex of approximately 120 kDa, corresponding to a 1:1 stoichiometry of EGFR_ex_ and EpEX and a second, weaker band of approximately 155 kDa, corresponding to a 1:2 stoichiometry of EGFR_ex_ and EpEX (Fig 3D; lane 6). The intensity of the approximately 120 kDa band was reduced upon further addition of EGF, and the second band of approximately 155 kDa disappeared (Fig 3D; lane 7), indicating a competitive binding of EpEX and EGF to EGFR_ex_.

Next, we assessed whether treatment of intact cells with EpEX-Fc as a ligand induces classical EGFR signaling pathways resulting in ERK1/2 and AKT phosphorylation. Treatment with EpEX-Fc (10 nM) induced phosphorylation of ERK1/2 in serum-starved FaDu, Cal27, and Kyse30 cells within minutes, which was inferior in intensity as compared to EGF treatment (1.8 nM) (Fig 3E). EpEX-Fc-induced activation of AKT at the indicated concentrations was similar to EGF, although time points of maximal induction varied between both ligands (Fig 3F). Treatment with therapeutic anti-EGFR antibody Cetuximab completely blocked EpEX-Fc- and EGF-mediated activation of ERK1/2 and AKT (Fig 3E and 3F), demonstrating an EGFR dependency of signaling. Activation of ERK1/2 and AKT phosphorylation by EpEX-Fc and EGF was further validated by immunofluorescence staining of FaDu and Cal27 cells. Imaging of ERK1/2 phosphorylation confirmed higher activation through EGF than EpEX-Fc, whereas AKT activation by EGF or EpEX-Fc was similar (Fig 3G). Owing to differential voltage adjustments, comparison across cell lines was not feasible. Furthermore, activation of ERK1/2 through EpEX-Fc and EGF in FaDu, Cal27, and Kyse30 cells was entirely blocked by an inhibitor of the upstream kinase MEK1 (AZD6244), whereas tyrosine kinase inhibitor (TKI) AG1478 entirely (Cal27) or partially (FaDu, Kyse30) blocked ERK1/2 activation by EpEX-Fc and EGF (Fig 3H). Hence, EpEX-Fc specifically activates phosphorylation of ERK1/2 through induction of EGFR signaling and MEK activity.

A requirement for EGFR expression to induce ERK1/2 by EpEX-Fc was analyzed in HEK293 cells, which do not express detectable amounts of EGFR [49]. Treatment of HEK293 cells with EpEX-Fc or EGF did not result in activation of ERK1/2 (Fig 3I). However, transient expression of EGFR restored signaling of EGF and EpEX-Fc to activate ERK1/2, confirming a specificity for EGFR for the observed effects of EpEX-Fc (Fig 3I). A possible involvement of full-length EpCAM in ERK1/2 activation by EpEX-Fc was addressed in clustered regularly interspaced short palindromic repeat/CRISPR-associated 9 (CRISPR-Cas9) *EPCAM*-knockout HCT8 cell lines [48]. HCT8 knockout cells entirely lacked EpCAM expression but retained EGFR levels comparable to wild-type and CRISPR-Cas9 control cell lines (Fig 3J). Treatment of EpCAM knockout with EpEX-Fc induced ERK1/2 phosphorylation within minutes (Fig 3K), demonstrating a requirement for EpEX but not for full-length EpCAM for ERK1/2 induction. Hence, EpEX-Fc is a ligand that induces specific activation of classical EGFR signaling pathways.

### Effects of EpEX-Fc and EGF are dosage dependent and differ in cellular outcome

**Fig 4:**
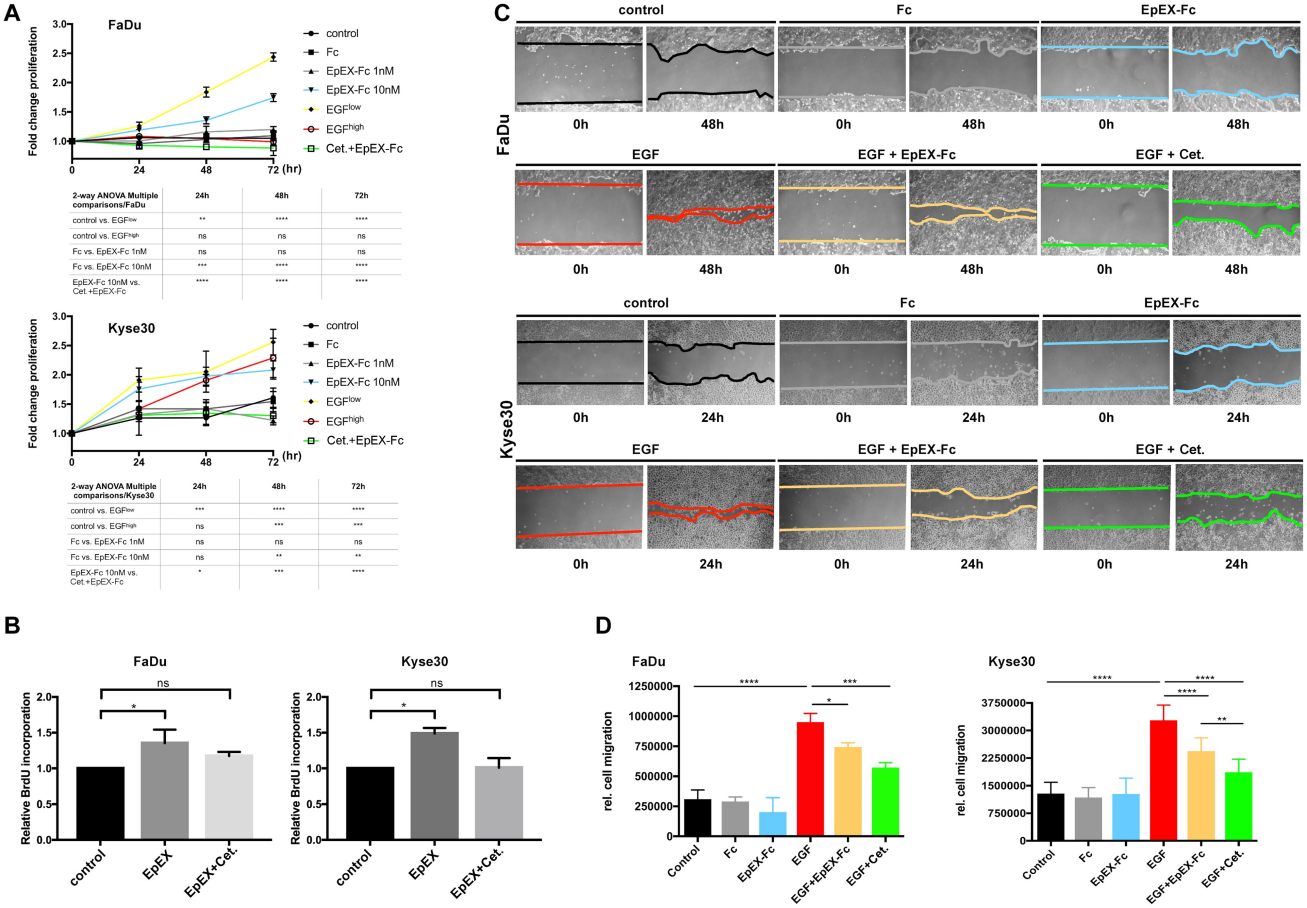
EpEX-Fc induces EGFR-dependent proliferation but inhibits high-dose EGF-induced EMT. (A) FaDu and Kyse30 cells were plated at equal numbers and treated with control media, low (1.8 nM) and high (9 nM) doses of EGF, low (1 nM) and high doses (10 nM) of EpEX-Fc, Fc (10 nM), or a combination of EpEX^high^ with Cetuximab (“Cet.”). Cell numbers were assessed after 24, 48, and 72 hr. Shown are means with SDs from *n* = 3 independent experiments. Two-way ANOVA with post hoc multiple testing and Bonferroni correction. * 0.05, ** 0.01; *** 0.001; **** 0.0001. Supporting data are compiled in S1 Data. (B) FaDu and Kyse30 cells were plated at equal numbers and treated with control media, EpEX (10 nM), or a combination of EpEX (10 nM) and Cetuximab (“Cet.”). BrdU incorporation was analyzed after 72 hr. Shown are means with SDs from *n* = 3 independent experiments. Two-way ANOVA with post hoc multiple testing and Bonferroni correction. * 0.05. Supporting data are compiled in S1 Data. (C) Relative migration of FaDu and Kyse30 cells was assessed in wound healing assays. FaDu, Kyse30, and Cal27 cells were either kept untreated (control) or were treated with Fc (10 nM), EpEX-Fc, EGF, EGF in combination with EpEX (10 nM), or EGF with Cetuximab (“Cet.”). Shown are representative micrograph pictures of cells after 24 hr (Kyse30) and 48 hr (FaDu) from *n* = 3 independent experiments. (D) Relative migration was quantified from representative micrographs and was normalized for proliferation indexes of each cell line. Shown are means with SDs from *n* = 3 independent experiments. One-way ANOVA with post hoc multiple testing and Bonferroni correction * *p*-value < 0.05, ** 0.01; *** 0.001. Supporting data are compiled in S1 Data. BrdU, bromodeoxyuridine; EGF, epidermal growth factor; EGFR, EGF receptor; EMT, epithelial-mesenchymal transition; EpEX, extracellular domain of EpCAM; Fc, fragment crystallizable region; ns, not significant; SD, standard deviation.

EpEX induces classical EGFR signaling pathways, which activate proliferation [22] but also promote EMT-characteristic phenotypic changes [50–53]. Therefore, we first compared effects of EGF and EpEX treatment on proliferation. Effects of EGF and EpEX-Fc on cell proliferation were assessed following treatment of FaDu and Kyse30 cells after 24, 48, and 72 hr. In order to delineate EGF and EpEX-Fc effects distinct from further growth factors, all cell lines were serum starved and maintained in the absence of serum throughout the experiment. Treatment of FaDu with low-dose EGF (1.8 nM) and high-dose EpEX (10 nM) induced a 2- and 1.5-fold increase in cell numbers, respectively, after 72 hr, whereas high-dose EGF (9 nM) did not induce proliferation (Fig 4A). EpEX-induced proliferation was entirely blocked upon cotreatment with Cetuximab (Fig 4A). Treatment with low-dose EGF, high-dose EGF, or high-dose EpEX-Fc (10 nM) induced a 2.5-, 2.2-, and 2-fold increase of Kyse30 cells, respectively, after 72 hr, whereas low-dose EpEX-Fc did not have significant mitogenic effect (Fig 4A). Similarly, EpEX-Fc-induced proliferation of serum-starved cells was blocked upon cotreatment with Cetuximab (Fig 4A). Proliferation-inducing effects of high-dose EpEX were additionally assessed through bromodeoxyuridine (BrdU) incorporation following treatment of serum-starved Kyse30 and FaDu cells. In line with cell counting results, treatment with high-dose EpEX (10 nM) induced a significant 50% and 30% increase in BrdU uptake after 72 hr in Kyse30 and FaDu cells, respectively. BrdU incorporation induced by EpEX was blocked by cotreatment with Cetuximab (Fig 4B).

Next, effects of high-dose EGF (9 nM) and EpEX (10 nM) on cell migration were assessed in scratch assays. EGF treatment at concentrations inducing EMT resulted in enhanced relative migration of serum-starved FaDu and Kyse30 cells, which was significantly reduced by cotreatment with EpEX-Fc and by Cetuximab (Fig 4C). Relative migration was quantified and corrected for proliferation rates, demonstrating substantially increased migration following EGF^high^ treatment and inhibitory effects of Cetuximab and EpEX-Fc (Fig 4D). Thus, EGF has dual capacities to induce proliferation and migration in HNSCC cell lines in a dosage-dependent manner, whereas EpEX-Fc induces proliferation at high concentration and counteracts EGF-induced migration.

### EpEX represses EGF-mediated EMT in HNSCC cell lines

**Fig 5:**
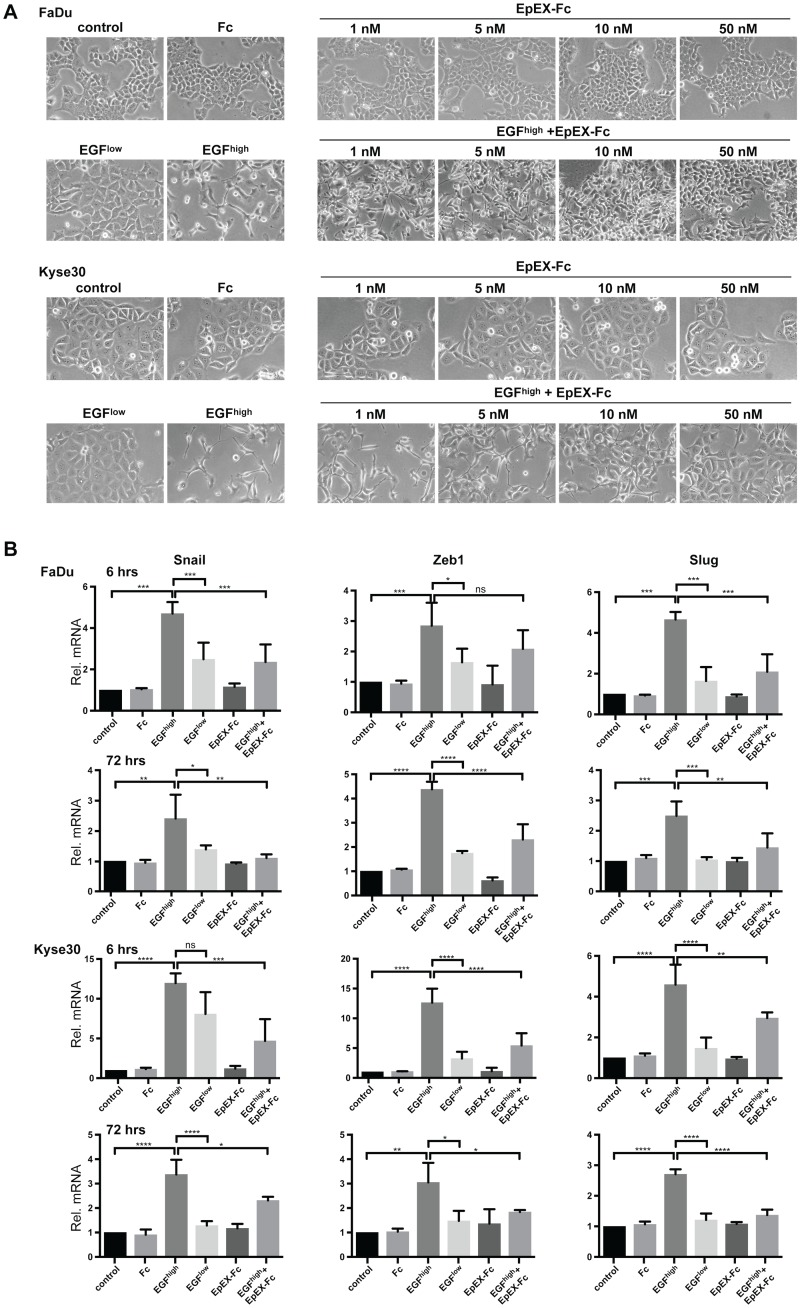
EpEX-Fc inhibits EGF-mediated EMT through repression of EMT-TF activation. (A) FaDu and Kyse30 cells were either kept untreated (control) or were treated with Fc (10 nM), EGF^low^ (1.8 nM), EGF^high^ (9 nM), EpEX-Fc (1–50 nM), or EGF^high^ in combination with increasing concentrations of EpEX-Fc (1–50 nM). Shown are representative micrograph pictures of cells after 48 hr (Kyse30) and 72 hr (FaDu) from *n* = 3 independent experiments. (B) FaDu and Kyse30 cells were either kept untreated (control) or were treated with Fc (10 nM), EGF^low^ (1.8 nM), EGF^high^ (9 nM), EpEX-Fc (10 nM), or EGF^high^ in combination with EpEX-Fc (10 nM). After 6 hr and 72 hr, mRNA transcript levels of Snail, Zeb1, and Slug were assessed by qRT-PCR with specific primers. Shown are means with SDs from *n* = 3 independent experiments performed in triplicates. One-way ANOVA with post hoc multiple testing and Bonferroni correction * *p*-value < 0.05, ** 0.01; *** 0.001. Supporting data are compiled in S1 Data. EGF, epidermal growth factor; EMT, epithelial-mesenchymal transition; EMT-TF, EMT transcription factor; Fc, fragment crystallizable region; ns, not significant; qRT-PCR, quantitative real-time PCR; SD, standard deviation; Zeb1, zinc finger E-box-binding homeobox 1.

Since EpEX partially inhibited EGF-induced migration, we next addressed whether this is associated with a capacity of EpEX to generally repress EGF-dependent EMT in HNSCC cell lines. Serum-starved FaDu, Kyse30, and Cal27 cells were treated with an increasing dose of EpEX-Fc (1–50 nM) or EGF at 1.8 and 9 nM for 48 to 72 hr. Treatment with an increasing amount of EpEX-Fc had no detectable effect on epithelial morphology (Fig 5A and S7A Fig). In contrast, treatment of FaDu and Kyse30 cells with high-dose EGF (9 nM), but not low dose (EGF 1.8 nM), reproducibly induced EMT with the generation of spindle-shaped cells and loss of cell–cell contact (Fig 5A). EMT induced by high-dose EGF was blocked upon simultaneous treatment with EpEX-Fc in a dose-dependent manner (Fig 5A). Treatment of Cal27 cells under the same conditions or with 2-fold-higher EGF concentration (18 nM) did induce neither EMT-related phenotypic changes nor loss of E-cadherin, which points at differences in cellular response (S7A and S7B Fig).

Next, regulation of E-cadherin, N-cadherin, vimentin, and EMT-TFs Snail, Zeb1, Slug, and Twist was assessed at the transcriptional levels after 6 and 72 hr following treatment. Treatment of cells with control media, Fc, or EpEX-Fc (10 nM) did not regulate the expression of any of the genes analyzed. Treatment of Kyse30 cells with EGF^high^ resulted in a transient up-regulation of vimentin mRNA after 6 hr, which was reduced to background after 72 hr (S7C Fig). E-cadherin, N-cadherin, and Twist expression was unaffected. Treatment of FaDu cells under the same conditions induced an early induction of N-cadherin after 6 hr that persisted until 72 hr (S7C Fig) and a decrease of E-cadherin and increase of Twist after 72 hr (S7C Fig). Inductions and repressions of gene expression following EGF treatment were all counteracted by cotreatment with high-dose EpEX. Treatment with EGF 9 nM resulted in substantial induction of Snail, Zeb1, and Slug in FaDu and Kyse30 cells at 6 hr and was maintained until 72 hr (Fig 5B). Simultaneous treatment of both cell lines with EGF (9 nM) and EpEX-Fc (10 nM) repressed Snail, Zeb1, and Slug induction (Fig 5B). Thus, EGF^high^ induces EMT changes with the recurrent expression of the EMT-TFs Snail, ZEB1, and Slug and partial loss of E-cadherin, whereas soluble EpEX-Fc counteracts EMT via repression of the abovementioned EMT-TF activation.

### Strength and duration of ERK1/2 activation integrates differential cellular fate

**Fig 6:**
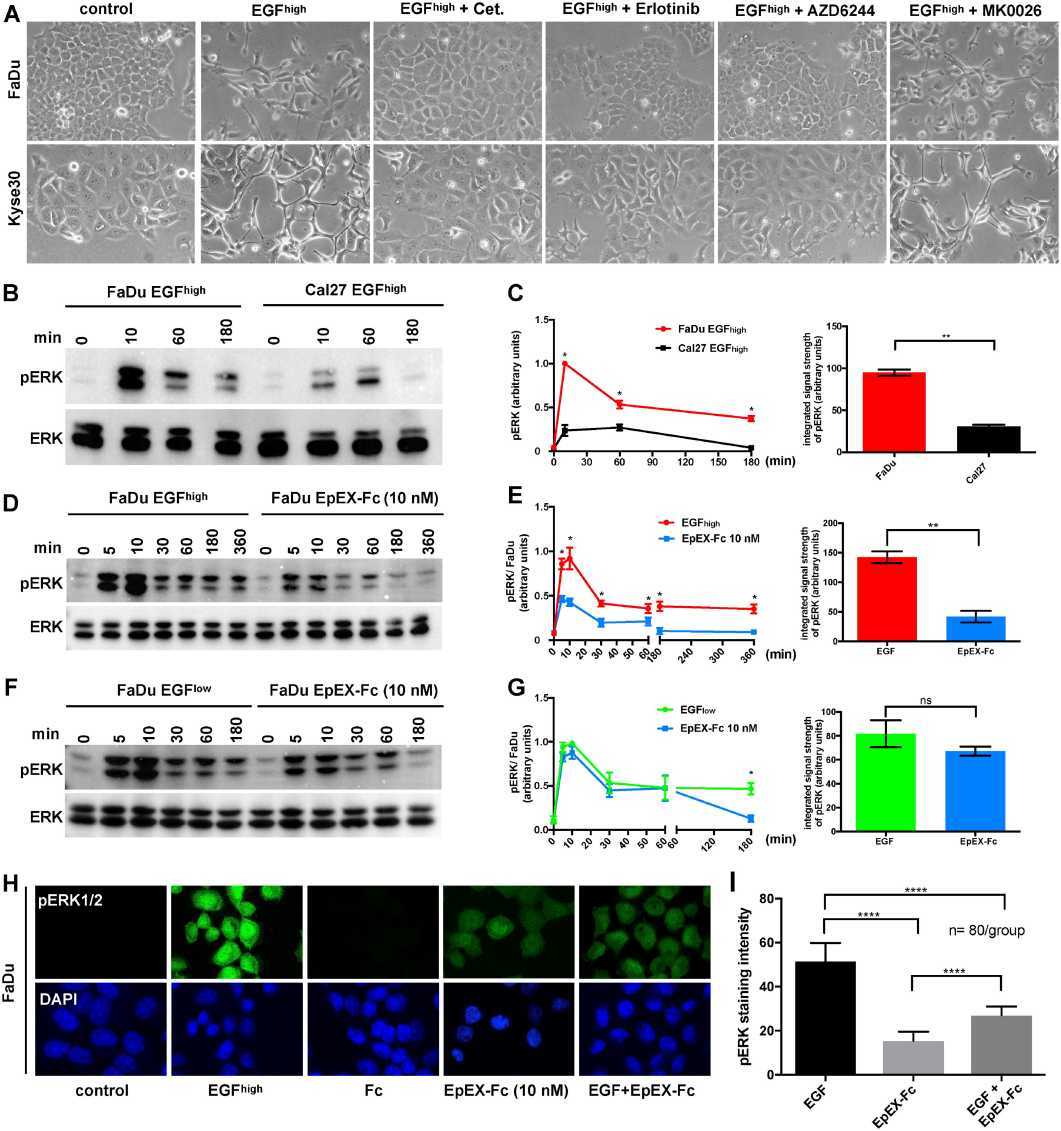
EpEX-Fc inhibits EGF-mediated EMT through modulation of ERK1/2 activity. (A) FaDu and Kyse30 cells were either kept untreated (control) or were treated with EGF (9 nM) and in combination with Cetuximab (“Cet.”), Erlotinib, AZD6244 (MEK1 inhibitor), and MK2206 (AKT inhibitor). Shown are representative micrograph pictures of cells after 48 hr (Kyse30) and 72 hr (FaDu) from *n* = 3 independent experiments. (B, C) FaDu and Cal27 cells were treated with EGF (9 nM) for the indicated time points, and ERK1/2 phosphorylation was assessed by immunoblotting. Levels of ERK1/2 were assessed in parallel. Shown are (B) representative results and (C) relative quantifications from *n* = 3 independent experiments. One-way ANOVA, multiple testing with Bonferroni correction. * *p*-value < 0.05, ** < 0.01. Supporting data are compiled in S1 Data. (D, E) FaDu cells were treated with EGF (9 nM) or EpEX-Fc (10 nM) for the indicated time points, and ERK1/2 phosphorylation was assessed by immunoblotting. Levels of ERK1/2 were assessed in parallel. Shown are (D) representative results and (E) relative quantifications from *n* = 3 independent experiments. One-way ANOVA, multiple testing with Bonferroni correction. * *p*-value < 0.05, ** < 0.01. Supporting data are compiled in S1 Data. (F, G) FaDu cells were treated with EGF (1.8 nM) or EpEX-Fc (10 nM) for the indicated time points, and ERK1/2 phosphorylation was assessed by immunoblotting. Levels of ERK1/2 were assessed in parallel. Shown are (F) representative results and (G) relative quantifications from *n* = 3 independent experiments. One-way ANOVA, multiple testing with Bonferroni correction. * *p*-value < 0.05. Supporting data are compiled in S1 Data. (H, I) FaDu and Cal27 cells were either kept untreated (control) or were treated with EGF (9 nM), Fc, EpEX-Fc (each 10 nM), or a combination of EGF and EpEX-Fc for 30 min. Phosphorylation of ERK1/2 was assessed by immunofluorescence laser scanning microscopy. Shown are (H) representative results and (I) relative quantifications from *n* = 3 independent experiments. One-way ANOVA, multiple testing with Bonferroni correction. **** *p*-value < 0.0001. Supporting data are compiled in S1 Data. pERK1/2: green, nuclei: blue (DAPI). EGF, epidermal growth factor; EMT, epithelial-mesenchymal transition; EpEX, extracellular domain of epithelial cell adhesion molecule; ERK1/2, extracellular signal–regulated kinase 1/2; Fc, fragment crystallizable region; ns, not significant; pERK1/2, phosphorylated ERK1/2.

So far, we have shown that EGF and EpEX-Fc both induce ERK1/2 and AKT phosphorylation, however, with differing intensities and cellular outcome. In order to shed light on signaling pathways implicated in EGF-dependent EMT and on mechanisms of EpEX-Fc-mediated inhibition, EMT-responsive FaDu and Kyse30 cells were treated with high-dose EGF in combination with Cetuximab, Erlotinib (TKI), AZD6244 (MEK1 inhibitor), and MK2206 (pan-AKT inhibitor). High-dose EGF induced pronounced EMT, which was completely blocked by Cetuximab, Erlotinib, and AZD6244 but not by AKT-inhibitor MK2206 in both cell lines, demonstrating that induction of ERK1/2 but not of AKT integrates EGF signals to mediate EMT (Fig 6A).

Since ERK1/2 was determined as the major mediator of EGF-induced EMT, we assessed potential differences in ERK1/2 activation in EMT-responsive FaDu versus nonresponsive Cal27 cells. Both cell lines were serum starved and treated with high-dose EGF (9 nM), and activating phosphorylation of ERK1/2 was assessed by immunoblotting after 10, 60, and 180 min. EGF treatment of FaDu cells induced a rapid and strong activation of ERK1/2, whereas Cal27 cells were only moderately and more transiently activated (Fig 6B). Signal quantification showed that the integrated pERK1/2 signal strength, incorporating intensity and duration, was 3-fold higher in FaDu cells than Cal27 cells (Fig 6C). Thus, integrated signal strength of pERK1/2 after EGF treatment represents a major switch in decision-making towards EMT induction.

Next, we assessed whether differential effects of EGF and EpEX-Fc on cell fate, i.e., EMT and proliferation, were related to the integrated signal strength of ERK1/2. FaDu cells were kept untreated or were treated with high-dose EGF (9 nM) or EpEX-Fc (10 nM), and phosphorylation levels of ERK1/2 were analyzed over time. ERK1/2 activation after EGF treatment was higher than with EpEX-Fc (Fig 6D). This translated in significantly enhanced pERK1/2 signal strength at all time points of treatment, with high-dose EGF compared to high-dose EpEX-Fc, and a 3-fold-enhanced integrated pERK1/2 signal strength (Fig 6E). In contrast, low-dose EGF (1.8 nM) and high-dose EpEX-FC (10 nM), which both trigger proliferation, induced a comparable average integrated pERK1/2 signal strength that was only significantly higher at late time points of EGF treatment (Fig 6F and 6G). Hence, high-dose EGF, but not high-dose EpEX-Fc, induces EMT through enhanced ERK1/2 activation intensity and duration.

Following, we addressed whether modulation of ERK1/2 activation is underlying the ability of EpEX-Fc to inhibit EMT induced by EGF. EMT-responsive FaDu cells were treated with high-dose EGF, high-dose EpEX-Fc, or a combination of both, and ERK1/2 phosphorylation was analyzed by immunofluorescence staining. Treatment with high-dose EGF induced a strong activation of pERK1/2, whereas activation by EpEX-Fc was more moderate (Fig 6H). Combinatorial treatment with EGF and EpEX-Fc resulted in reduced ERK1/2 activation, compared to only EGF (Fig 6H). Quantification of pERK1/2 disclosed a 3-fold-reduced ERK1/2 activation by EpEX-Fc compared to EGF and a 2-fold reduction of activity upon combinatorial treatment with EGF and EpEX-Fc, compared to strong induction by EGF alone (Fig 6I). Thus, EpEX-Fc potently inhibits EGF-dependent EMT through modulation of the ERK1/2 activation.

### EGFR and EpCAM levels control EMT induction in vitro and mimic the in vivo situation

**Fig 7:**
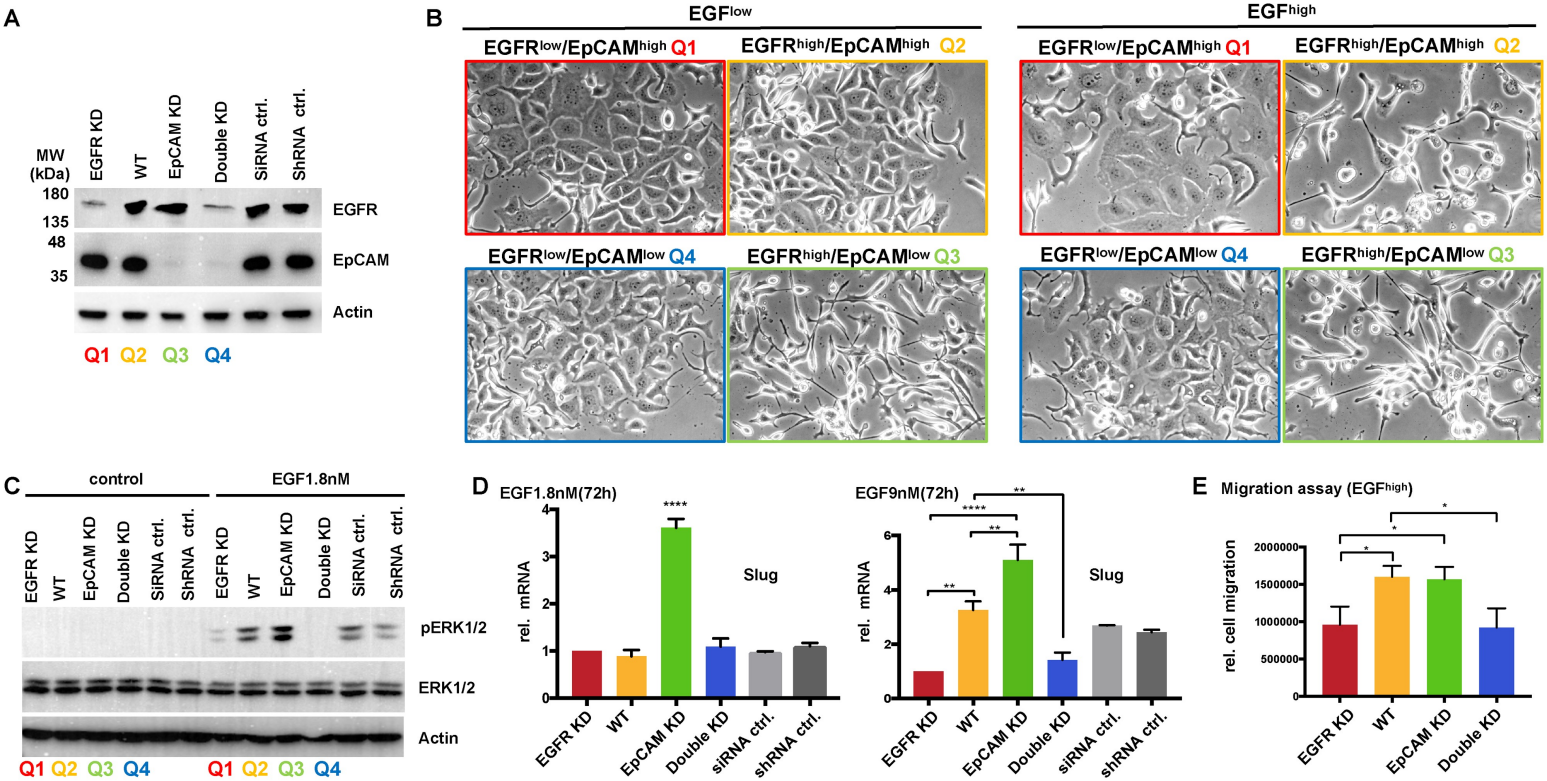
EGFR and EpCAM levels are molecular determinants of EMT induction, ERK activation, and migration. (A) Kyse30 cells were transfected with EGFR-specific siRNAs (pool of *n* = 4 siRNAs) (EGFR KD), siRNA controls (siRNA ctrl), EpCAM-specific shRNA (EpCAM KD) [54], control shRNA (shRNA ctrl), and a combination of EGFR siRNA and EpCAM shRNA (double KD). Expression of EGFR and EpCAM was assessed by immunoblotting with specific antibodies. Equal loading was confirmed by detecting actin levels. Clinical quadrants’ equivalents are indicated. Shown are representative results from *n* = 3 independent experiments. (B) Quadrant 1 to 4 equivalents of Kyse30 cell variants were treated with EGF^low^ (1.8 nM) and EGF^high^ (9 nM). Cell morphology was assessed after 72 hr. Shown are representative images from *n* = 3 independent experiments. (C) Quadrant 1 to 4 equivalents of Kyse30 cell variants were treated with EGF^low^ (1.8 nM) for the indicated time points, and ERK1/2 phosphorylation was assessed by immunoblotting. Levels of ERK1/2 and actin were assessed in parallel. Shown are representative results from *n* = 3 independent experiments. (D) Quadrant 1 to 4 equivalents of Kyse30 cell variants were either kept untreated (control) or were treated with EGF^low^ (1.8 nM). After 72 hr, mRNA transcript levels of Slug were assessed by qRT-PCR with specific primers. Shown are means with SDs from *n* = 2–3 independent experiments performed in triplicates. One-way ANOVA with post hoc multiple testing and Bonferroni correction * p-values < 0.05, ** 0.01; *** 0.001, **** 0.0001. Supporting data are compiled in S1 Data. (E) Quadrant 1 to 4 equivalents of Kyse30 cell variants were treated with EGF^high^ (9 nM) and subjected to a scratch assay. Relative migration was quantified from representative micrographs of each cell line. Shown are means with SDs from *n* = 3 independent experiments. One-way ANOVA with post hoc multiple testing and Bonferroni correction * p-value < 0.05. Supporting data are compiled in S1 Data. EGF, epidermal growth factor; EGFR, EGF receptor; EpCAM, epithelial cell adhesion molecule; EMT, epithelial-mesenchymal transition; ERK, extracellular signal–regulated kinase; KD, knockdown; pERK1/2, phosphorylated ERK1/2; qRT-PCR, quantitative real-time PCR; SD, standard deviation; shRNA, short hairpin RNA; siRNA, small interfering RNA; WT, wild type.

EGFR and EpCAM levels correlated with the clinical outcome of HNSCCs (see Fig 1 and S1 Fig). In order to address the impact of EGFR and EpCAM expression on tumor cell behavior at the mechanistic level, we established an in vitro mimic of the clinical situation. Kyse30 cells were treated with an EGFR-specific small interfering RNA (siRNA) pool, an EpCAM-specific shRNA, and the cognate controls. Double knockdown of EGFR and EpCAM was performed in EpCAM-knockdown Kyse30 cells with an EGFR-specific siRNA pool. EGFR and EpCAM expression levels were confirmed by immunoblotting (Fig 7A). Based on these expression levels, EGFR-knockdown Kyse30 cells mimicked quadrant 1 of the clinical cohort (EGFR^low^/EpCAM^high^). Wild-type Kyse30 cells (EGFR^high^/EpCAM^high^), EpCAM-knockdown Kyse30 cells (EGFR^high^/EpCAM^low^), and double-knockdown Kyse30 cells (EGFR^low^/EpCAM^low^) mimicked quadrant 2, 3, and 4, respectively.

Next, all cell lines were treated with EGF^low^ (1.8 nM) and EGF^high^ (9 nM), and induction of EMT was assessed based on the cell morphology at 72 hr. In EGFR^high^/EpCAM^high^ wild-type cells (Q2 equivalent), we confirmed the induction of EMT following treatment with EGF^high^ but not EGF^low^ (Fig 7B). Knockdown of EGFR in Kyse30 cells (EGFR^low^/EpCAM^high^, Q1 equivalent) abolished EMT induction at both EGF concentrations. Double knockdown of EGFR and EpCAM (EGFR^low^/EpCAM^low^, Q4 equivalent) also resulted in a lack of EMT induction following EGF treatment (Fig 7B). Knockdown of EpCAM in the presence of high levels of EGFR (EGFR^high^/EpCAM^low^, Q3 equivalent) resulted in a 5-fold-reduced level of EGF required for the induction of EMT, with strong EMT induction at EGF^low^ concentrations (Fig 7B).

We have determined modulation of ERK1/2 activation strength as a major molecular switch to regulate proliferation versus EMT (see Fig 6). Therefore, serum-starved Kyse30 cell variants representing clinical quadrants 1–4 were treated with EGF^low^ (1.8 nM), and activating phosphorylation of ERK1/2 was assessed by immunoblotting. In EGFR^high^/EpCAM^high^ wild-type cells (Q2 equivalent), EGF^low^ induced intermediate ERK1/2 phosphorylation, which was strongly reduced in EGFR-knockdown Kyse30 cells (EGFR^low^/EpCAM^high^, Q1 equivalent) (Fig 7C). Double knockdown of EGFR and EpCAM (EGFR^low^/EpCAM^low^, Q4 equivalent) entirely abolished ERK1/2 activation (Fig 7C). Knockdown of EpCAM in the presence of high levels of EGFR (EGFR^high^/EpCAM^low^, Q3 equivalent) resulted in enhanced ERK1/2 activation (Fig 7C). This increased activation of ERK1/2 in EGFR^high^/EpCAM^low^ Kyse30 cells was paralleled by induction of EMT-TF Slug following EGF^low^ treatment, whereas all other cells variants did not induce mRNA expression of Slug under these conditions (Fig 7D). Treatment of all Kyse30 cell variants with EGF^high^ induced Slug expression in EGFR^high^/EpCAM^high^ wild-type cells (Q2 equivalent), which was significantly reduced following EGFR knockdown (Q1 and 4 equivalents) and enhanced following EpCAM knockdown (Q3 equivalent) (Fig 7D). Accordingly, EGF^high^ treatment induced a higher relative migration rate in quadrant 2 and 3 mimics (wild-type and EpCAM-knockdown Kyse30 cells) than in quadrant 1 and 4 mimics (EGFR-knockdown and EGFR/EpCAM-double-knockdown Kyse30 cells) (Fig 7E).

Hence, loss of EGFR abolished—whereas loss of EpCAM facilitated—EGF-induced EMT through modulation of ERK activation and Slug expression. These differences further impacted the cellular behavior, with increased migration rates in quadrant 2 and 3 mimics.

### pERK1/2 and Slug define HNSCC patients with poor outcome

**Fig 8:**
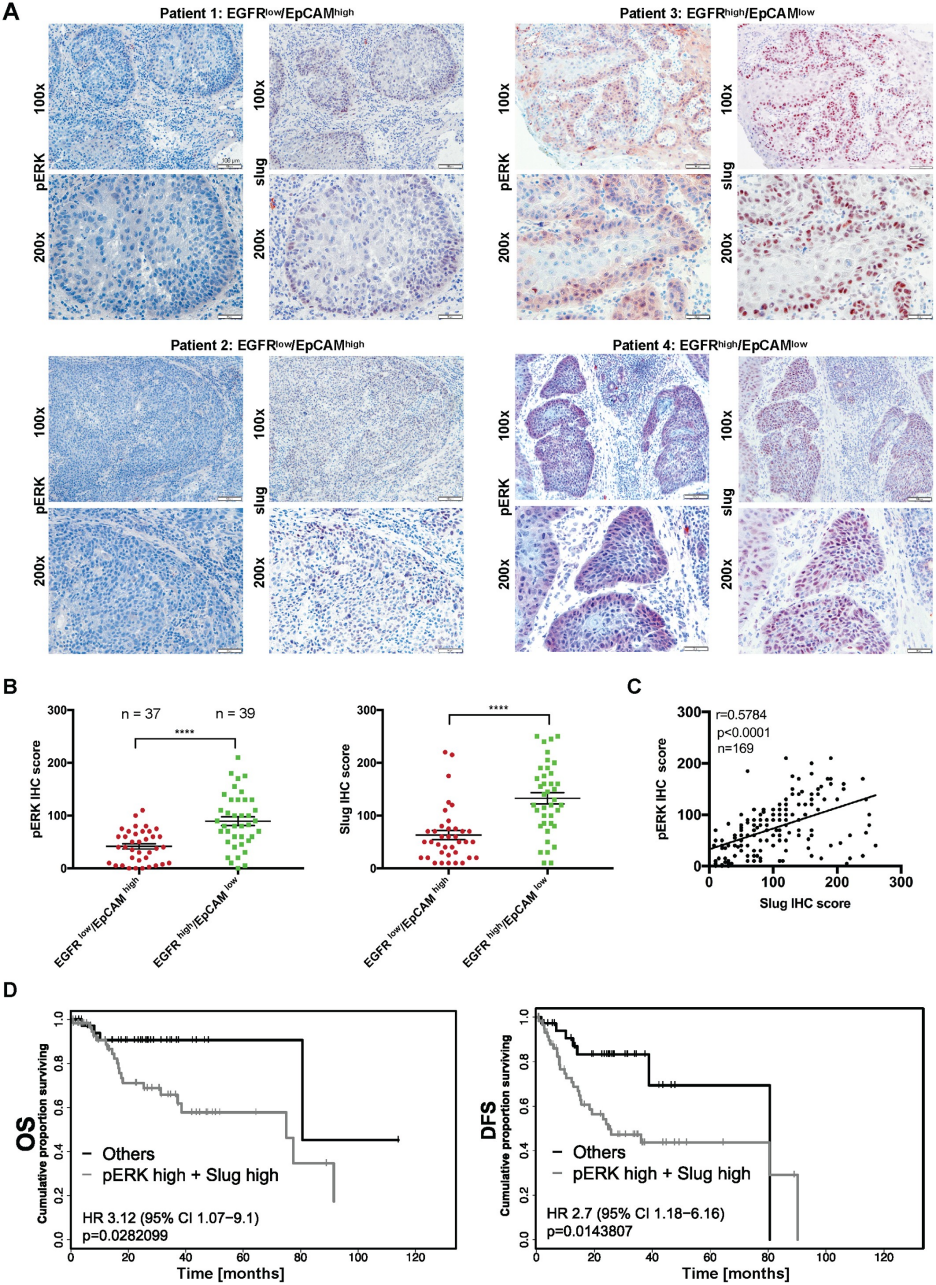
pERK and Slug are coexpressed and predict poor survival of HNSCC patients. (A) HNSCC tumors were stained for the expression of pERK1/2 and Slug in consecutive cryosections. Shown are 2 examples each of EGFR^low^/EpCAM^high^ (patients 1 and 2), EGFR^high^/EpCAM^low^ (patients 3 and 4), and HNSCCs at 100× and 200× magnification. (B) IHC scores of pERK and Slug were compared in EGFR^low^/EpCAM^high^ (*n* = 37) and EGFR^high^/EpCAM^low^ (*n* = 39) HNSCCs. Shown are IHC score values with mean (lines) and Student *t* test. ** p-value < 0.01. Supporting data are compiled in S1 Data. (C) IHC scores of pERK and Slug were compared in a Spearman correlation with r-value and *p*-value for the entire HNSCC cohort (*n* = 169/180). Supporting data are compiled in S1 Data. (D) Patients with EGFR and EpCAM expression levels <125 or >175 were included in the survival analysis. OS (*n* = 98 patients) and DFS (*n* = 97 patients) were stratified according to pERK and Slug expression (cutoff threshold IHC score median). Patients with expression of pERK1/2 and/or Slug >175 are considered as high expressers (pERK high + Slug high) and were compared to all remaining patients. OS and DFS are represented as Kaplan-Meier survival curves with *p*-values, HRs, and CIs. Supporting data are compiled in S1 Data. CI, confidence interval; DFS, disease-free survival; EGFR, epidermal growth factor receptor; EpCAM, epithelial cell adhesion molecule; HNSCC, head and neck squamous cell carcinoma; HR, hazard ratio; IHC, immunohistochemistry; OS, overall survival; pERK, phosphorylated extracellular signal–regulated kinase.

As reported for breast cancer, high ERK1/2 activity results in enhanced transcription and expression of Slug to foster cell migration [55]. Slug in turn was the only EMT-TF that was up-regulated in the single-cell transcriptomic pEMT-signature of HNSCCs (termed SNAIL 2 in that publication) [16] and appears as an early EMT-inducing factor compared to other EMT-TFs [56]. Based on these reports and on the findings from our own in vitro study, we addressed whether ERK1/2 and Slug levels in vivo reflected the abovementioned regulation mechanisms in HNSCCs. EGFR^low^/EpCAM^high^ (*n* = 37) and EGFR^high^/EpCAM^low^ specimens (*n* = 39) were stained for the expression of pERK1/2 and Slug in consecutive sections. EGFR^high^/EpCAM^low^ specimens were characterized by high average pERK1/2 and Slug expression, whereas EGFR^low^/EpCAM^high^ specimens displayed low to moderate expression of both antigens (Fig 8A). Quantification using IHC scoring revealed an average 2.14-fold-enhanced level of ERK1/2 activation and an average 2.1-fold increase in Slug expression in EGFR^high^/EpCAM^low^ tumors compared to EGFR^low^/EpCAM^high^ tumors (Fig 8B).

In order to test overall correlations of pERK1/2 and Slug, consecutive sections of all available patients’ specimens of the HNSCC cohort (*n* = 169/180) were stained for pERK1/2 and Slug expression. Expression levels of both proteins were quantified using the IHC scoring system. Spearman correlation analysis of IHC scores confirmed a robust positive correlation of pERK1/2 and Slug expression in the HNSCC cohort (Fig 8C, r = 0.5784, *p* < 0.0001). Concurrent expression of pERK1/2 and Slug at the edges of tumor areas was observed (Fig 8A) and corroborated the preferential positioning of EMT cells at the leading edges of HNSCCs [16]. Next, expression levels of pERK1/2 and Slug were tested for their prognostic value. To do so, patients with EGFR and EpCAM IHC scores below 125 or above 175 were included. High expression of pERK1/2 and/or Slug (cutoff threshold IHC score median) defined HNSCCs with poor OS (*n* = 98) and DFS (*n* = 97) (Fig 8D). Hence, EGF/EGFR/pERK1/2/Slug represents a signaling axis that impacts cell differentiation towards EMT and defines HNSCC patients with poor clinical performance.

## Discussion

HNSCC patients have dismal clinical outcome, with overall death rates above 55% at 5 y [2]. Recent publications disclosed an outstandingly high genetic and cellular heterogeneity of HNSCCs at the inter- and intratumoral level that may account for therapy resistance and poor clinical performance [8–11,16]. Especially, shifts towards mesenchymal phenotypes of carcinoma cells emerged as central to metastases formation, therapy resistance, and, ultimately, poor prognosis in several carcinoma entities [4,5,57–60], including HNSCCs [16].

EGFR is currently the major therapeutic target in palliative treatment regimens for recurrent and metastatic HNSCCs and colon and lung cancer [17–20,26,61–65], with signaling capacities to induce a broad range of cellular outcomes such as proliferation and EMT [15,24–27,53,65–68]. EpCAM in turn has been described as a cell adhesion molecule and, more recently, as a signaling membrane protein that regulates cell proliferation and differentiation in cancer and stem cells [38,39,43,69–72]. EpCAM defines the degree of epithelial differentiation of HNSCCs, and its expression at the single cell RNA-sequencing level was opposite to genes composing a pEMT signature including vimentin and Slug [16]. Loss of EpCAM was observed during EMT [73,74], but a causal role in EMT is not fully understood [54,73,75–79]. Recently, Hsu and colleagues provided a link between EGF-induced EMT and RIP of EpCAM in the endometrial carcinoma cell line RL95-2. In their model, EGF/EGFR signaling induced a complete loss of EpCAM because of cleavage that released EpICD. Together with lymphoid enhancer-binding factor 1 (Lef-1), EpICD then served as an inductor of EMT upon nuclear translocation and activation of an EMT gene program [36]. Although the presented mechanism would theoretically provide a compelling molecular basis for EGF/EGFR-dependent EMT and concurrent loss of EpCAM, unexpected results from the use of γ-secretase inhibitors raised questions on the actual molecular mechanism [36,80]. In the present study, RIP of EpCAM through EGF/EGFR signaling could neither be observed in an array of carcinoma cell lines nor be reproduced in RL95-2 endometrial carcinoma cells, independently of time points and EGF concentrations used (Fig 2). We conclude that EGF-induced RIP of EpCAM is neither a common nor a frequent mechanism in carcinoma cell differentiation along the EMT. The molecular background for such contradicting findings on the role(s) of EpCAM in EMT regulation has, to the best of our knowledge, not been elucidated yet. In accordance with such discrepancies, high expression of EpCAM is frequently associated with poor clinical outcome of breast, colorectal, pancreatic, and nasopharyngeal carcinomas and ovarian and bladder cancers [79,81–89] but with a good prognosis of colonic, gastric, and renal cancer [90–92] and of HNSCCs, as shown in the present study.

**Fig 9:**
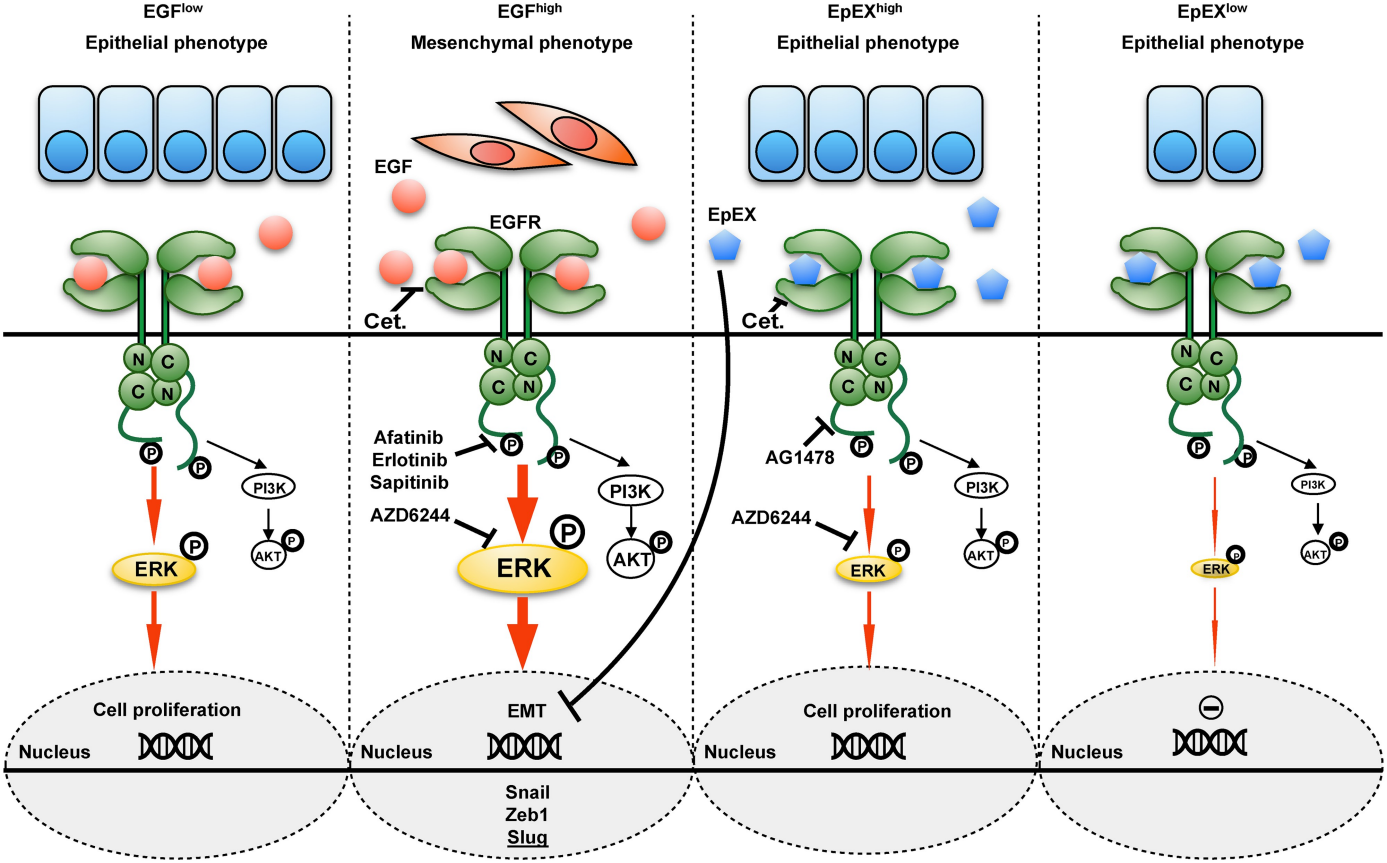
Schematic representation of EGF and EpEX cross talk at the EGFR. Low-dose EGF induces EGFR activation that results in intermediate ERK1/2 phosphorylation and enhanced cell proliferation (left panel). High-dose EGF induces EGFR activation that results in strong ERK1/2 phosphorylation and induction of EMT, including EMT-TFs Snail, Zeb1, and Slug (center-left panel). High-dose EpEX induces EGFR activation that results in intermediate ERK1/2 phosphorylation and enhanced proliferation (center-right panel); low-dose EpEX has no measurable effect on proliferation (right panel). EGF, epidermal growth factor; EGFR, EGF receptor; EMT, epithelial-mesenchymal transition; EMT-TF, EMT transcription factor; EpEX, extracellular domain of EpCAM; ERK1/2, extracellular signal–regulated kinase; PI3K, phosphoinositide 3-kinase; Zeb1, zinc finger E-box-binding homeobox 1.

Alternatively to Hsu and colleagues, we describe a novel functional cross talk of EGFR and EpCAM that regulates proliferation and EMT (Fig 9), which provides a molecular basis for differences in clinical outcome of subgroups of HNSCC patients. EGFR^high^ HNSCCs were associated with poor OS in our cohort and in the HNSCC TCGA cohort [9], including both HPV-negative subcohorts. Combination of EGFR and EpCAM as biomarkers for HNSCCs demonstrated that EGFR^low^/EpCAM^high^ HNSCCs were characterized by improved OS and DFS, whereas EGFR^high^/EpCAM^low^ tumors were characterized by strongly reduced OS and DFS (Fig 1). A tendency of an enrichment of oropharyngeal carcinoma associated with chronic HPV infection within the EGFR^low^/EpCAM^high^ group conforms with better survival [2,29,32,93–96]. However, analyses of HPV-negative patients within the LMU cohort confirmed results of the full cohort, arguing that the observed disparities in clinical outcome were not dependent upon the HPV status. Furthermore, effects were not related to differing sublocalizations of HNSCC specimens in our cohort. Analyses of the more abundant group of patients of oropharyngeal carcinomas, which are generally associated with improved survival, still demonstrated a significantly improved OS and DFS of EGFR^low^/EpCAM^high^ versus EGFR^high^/EpCAM^low^ tumors. Hence, an EGFR/EpCAM cross talk might generally be instrumental in the regulation of malignant differentiation in HNSCCs and thus impact clinical outcome.

Functionally, we demonstrate that activation of EGFR through EGF in HNSCC cells results in a dosage-dependent dual capacity to activate proliferation or EMT through differential ERK1/2 activation strength and duration. Intermediate ERK1/2 activation correlated with induction of proliferation, whereas strong and sustained ERK1/2 activation was required to induce EMT (Fig 9). This is in accordance with reported differential integration of ERK signaling to modulate cell fates in PC12 and 3T3 cells [97,98]. Inhibitors of AKT did not impact EGF-induced EMT, defining ERK1/2 as a major integrator of EGFR-mediated signals into proliferation or EMT. In line with a role of ERK1/2 in EGF-mediated EMT, nonresponsive cells were characterized by substantially lower ERK1/2 induction rates. A central role of ERK1/2, rather than AKT, in EGF-induced EMT induction conforms with earlier reporting [50–52,99] but is in contradiction to the published role of AKT in prostate cancer cell lines [53], nasopharynx carcinoma [79], and mammary MCF7 cells [100]. Direct involvement of ERK1/2 in EMT regulation has been reported to function through transcriptional silencing of E-cadherin expression [52]. Despite a loss of E-cadherin protein expression in both HNSCC lines FaDu and Kyse30 following EGF treatment, E-cadherin mRNA levels remained unaffected in Kyse30 cells, suggesting additional posttranslational effects of EGF/EGFR/ERK1/2 on E-cadherin expression. Additionally, EGF/EGFR/ERK1/2 robustly affected the expression of Snail, Zeb1, and Slug in all cell lines addressed, but not as consistently Twist, N-cadherin, and vimentin (Figs 2, 4 and S7 Fig). Selective regulation of EMT-associated genes has been reported and contributes to the fluxionary and gradual nature of EMT in cancer [4,60,101].

Our results further provide a molecular mechanism in support of ERK1/2 as potential target(s) in HNSCCs (Figs 3–8), the inhibition of which was reported to enhance antitumor therapy in clinical approaches [102,103]. Induction of EMT through EGF/EGFR/ERK1/2 entailed functional consequences, as cells were equipped with enhanced migration capacity and might thereby impact local and distant tumor cell dissemination. Induction of migration and invasion through EMT might generate multifocal nests of therapy-resistant cells that have delaminated from the primary tumor, evade surgery, and could give rise to subsequent relapse. Accordingly, EMT was implicated in the generation of tumor cells with stem-like capacity (CSCs), which represent the source of tumor recurrences and metastases [6,15,104]. Phenotypic plasticity along the EMT allows subpopulations of HNSCC-CSC to switch between epithelial/proliferative and mesenchymal/migratory/invasive states [105]. Post-EMT HNSCC-CSC, characterized as CD44^high^/EpCAM^low/neg.^/CD24^pos.^, have enhanced resistance towards therapeutic drugs [106].

In accordance with a central role for pERK1/2 in induction of EMT in HNSCCs, we demonstrate a correlation of pERK1/2 with the EMT-TF Slug within clinical samples and with substantially decreased OS and DFS (Fig 8). Hence, the elucidation of a novel EGFR/EpCAM cross talk in HNSCCs additionally defined pERK1/2 and Slug as biomarkers for the stratification of HNSCC patients. Induction of Slug through the effect of pERK1/2 has been reported for breast cancer migration [55], and Slug emerged as the only EMT-TF significantly associated with a pEMT signature in single cells of HNSCCs [16]. In analogy to the published intratumoral localization of pEMT-signature genes LAMB3 and LAMC2 [16], pERK1/2 and especially Slug were frequently expressed in cells of the leading edge of tumor areas in our cohort. Thus, modulation of differential strength and quality of EGFR downstream signaling by EGF and EpEX might considerably impact key processes of local invasion and eventually recurrence. Increased activation of EGFR at the edges of tumor areas to induce EMT might be fulfilled by cancer-associated fibroblasts, myeloid-derived cells [107], or, as recently reported, endothelial cells secreting EGF and inducing EMT- and stem-like properties in HNSCCs [108].

Using a cellular system, we recapitulated in vitro the distribution of EGFR and EpCAM expression observed in clinical samples. By doing so, we corroborated a regulatory potential of EGFR and EpCAM in the induction of EMT in HNSCC cells. Knockdown of EGFR expression inhibited EMT induction by EGF and was paralleled by a lack of activation of ERK1/2 and Slug and by reduced migration, hence demonstrating a central role of EGFR. Knockdown of EpCAM expression facilitated EGF-mediated induction of EMT, with EGF concentrations required for activation of EMT, pERK1/2, and Slug that were reduced 5-fold. We therefore suggest that EGFR and EpCAM levels in vivo impact the strength of ERK and Slug activation through stimulation of EGFR. Reduced levels of EpCAM facilitate induction of EMT through a release from the competitive effects of EpEX on EGF and a resulting decrease in EGF concentrations required for strong ERK1/2 activation towards EMT. Hence, EpCAM emerges not only as a surrogate marker for epithelial differentiation of HNSCCs [16] but also as a modulator of epithelial differentiation with functional implication in tumors and stem cells [70]. High expression of EpCAM can be positive for treatment outcome based on its role in cell–cell adhesion [69,109], in proliferation [38,39], in endomesodermal differentiation [70], and, as first described here, as a regulatory molecular determinant of tumor cell differentiation along the EMT axis through modulation of EGFR-dependent pERK1/2.

Furthermore, we provide evidence that the soluble ectodomain EpEX, which is produced upon RIP of EpCAM [39,48,110] including HNSCC cell lines (Fig 3), is a regulatory ligand of EGFR that induces ERK1/2 and AKT signaling (Fig 9). The definition of EpEX as a ligand for EGFR is in line with recent reports on the activation of EGFR signaling after treatment with EpEX in mouse embryonic fibroblasts and in colon carcinoma cell lines [71,111]. In colon carcinoma cells, EpEX induced mild proliferation and regulated proteolysis of intact EpCAM molecules through the activation of ADAM17 and γ-secretase [111]. However, experimental evidence for the binding of EpEX to EGFR was lacking. Additionally, activation of migration of colon carcinoma cells through EpEX is in contradiction with our findings in Kyse30 and FaDu HNSCC cell lines.

Serum levels of EpEX in tumor patients revealed low [46,110,112–114] but represent systemic levels, which suggests substantially higher intratumoral levels at the interface of tumor cells, where EpEX is actively shed [115]. Hence, production of EpEX by carcinoma cells could locally impact the regulation of EGFR-dependent proliferation and EMT. Activation of signaling by EpEX was specific, as it depended on the expression of EGFR and was blocked by Cetuximab, TKIs, and ERK- and AKT-specific inhibitors. Unlike EGF, EpEX induces EGFR and ERK1/2 less potently and eventually promotes mild proliferation rather than EMT. Cotreatment of HNSCC cells with EpEX led to a dose-dependent repression of EGF-mediated EMT, which was accompanied by reduction of ERK1/2 activation and Snail, Zeb1, and Slug transcription. We suggest that EpEX directly binds EGFR, as shown by cross-linking of recombinant proteins, but activates ERK1/2 less efficiently than EGF. Thereby, EGF and EpEX compete for binding to EGFR_ex_, and EpEX modulates the strength of EGFR signaling to pERK1/2/Slug, repressing EMT in cancer cells (Figs 5 and 9).

Inhibition of EGF-mediated ERK activation by full-length EpCAM has been reported in carcinoma cells, including breast cancer lines [73]. Down-regulation of EpCAM resulted in increased ERK activity following treatment with EGF and enhanced Slug expression. Oppositely, forced expression of EpCAM was shown to reduce ERK activation and Slug expression [73]. In concordance, knockdown of EpCAM in Kyse30 cells resulted in enhanced ERK1/2 activation by EGF in our in vitro experiments. A priori, our findings of an activating effect of EpEX on ERK activation are contradicting the report by Sankpal and colleagues [73]. However, our findings demonstrate that EpEX can compete with the strong activation of ERK, Slug, and eventually EMT following activation of EGFR by EGF (Figs 5–8). Hence, in addition to an inhibitory role of EpEX on the strong activation of ERK1/2, Snail, Zeb1, and Slug by EGF, EpEX has itself an intermediately activating effect on ERK activation in serum-starved HNSCC lines that results in proliferation. Work by Lin and colleagues in colon cancer cells demonstrated a function of EpCAM in the activation of pluripotency genes and EMT regulators [42], which is contradictory to our findings. Once again, cellular systems and especially the molecules addressed are differing. Whereas Lin and colleagues concentrated on EpCAM in the regulation of transcription factors, our study addresses the cross talk of EGFR with EpCAM—more specifically, with the soluble ectodomain EpEX. From our cellular system, we conclude that EpEX binding to EGFR does not induce EMT-TFs or EMT but can induce proliferation and compete with EGF to hamper EMT induction. Thus, a cross-regulatory role of EGFR and EpCAM appears as a general regulatory mechanism that is instrumental in carcinoma cells and includes negative and positive feedback loops to control ERK1/2 and, ultimately, cell fate.

In summary, we describe a novel molecular cross talk of EGFR and EpCAM that provides a rationale for substantial differences in survival of HNSCC patients. Stratification of HNSCC patients based on EGFR, EpCAM, pERK1/2, and Slug expression levels represents a promising tool to define patients at increased risk of clinical relapse, with the future aim to improve therapeutic intervention including EGFR and EpCAM as targets.

## Materials and methods

### Human samples and ethics statement

The LMU of Munich, Germany, HNSCC cohort included tumor specimens from 180 patients. Distant normal mucosa was available for 87 patients. Clinical samples were obtained after written informed consent during routine surgery, based on the approval by the ethics committee of the local medical faculties (Ethikkommission der Medizinischen Fakultät der Ludwig-Maximilians-Universität; #087–03; #197–11; #426–11) and in compliance with the WMA Declaration of Helsinki and the Department of Health and Human Services Belmont Report.

### Cell lines and treatments

FaDu, Kyse30, Cal27, Cal33, HCT8, RL95-2, HEK293, Du145, MCF7, and MDA-MB-231 cell lines were obtained from ATCC and DSMZ and were confirmed by STR typing (Helmholtz Center, Munich, Germany). Kyse30-shRNA lines were described in [54]. Kyse30 and HCT8 cells were stably transfected with EpCAM-YFP fusion in the 141-pCAG-3SIP vector as described [39,48]. Cells were maintained in RPMI 1640 or DMEM, 10% FCS, 1% penicillin/streptomycin, in a 5% CO_2_ atmosphere at 37 °C. Treatment with EGF (PromoCell PromoKine, Heidelberg, Germany), EpEX-Fc, Cetuximab (Merck Serono, Darmstadt, Germany, 10 μg/mL), AZD6244 (Selleckchem, Munich, Germany; 1 μM), AG1478 (Selleckchem, 10 μM), Erlotinib (Selleckchem, 1 μM), MK2206 (Selleckchem, 1 μM), ß-lactone (Santa Cruz, Heidelberg, Germany, 50 μM) was conducted in medium. Recombinant EpEX-Fc was produced as described [48]. Briefly, HEK293 cells were stably transfected with human EpEX-Fc fusion in the 141-pCAG-3SIP vector and cultured in ultralow IgG DMEM for EpEX-Fc purification. EpEX-Fc was purified from supernatant of transfected cells after 3–5 d of culture according to the protocol by Savas and colleagues [116]. Recombinant Fc was purchased from Jackson ImmunoResearch, Baltimore, MD, United States.

### Migration assay, cell proliferation, BrdU incorporation

Migration was assessed as described [54]. Quantification of scratches was performed using ImageJ and MRI wound healing tool. Relative migration was adjusted for proliferation rates. For cell proliferation, 1 × 10^5^ cells were plated in 12-well plates before treatment. At indicated time points, cells were counted in an EVE automatic cell counter (NanoEntek, VWR, Munich, Germany). BrdU proliferation assay was performed with the Cell Proliferation ELISA BrdU kit (Roche, Penzberg, Germany) following the manufacturer’s protocol. Cells were plated at a density of 5,000 cells/well into a 96-well plate before treatment. After serum starvation, cells were treated as indicated in figure legends for 72 hr. BrdU (10 μM) was then added to the cells and incubated for 12 hr. BrdU was detected with a peroxidase-labeled anti-BrdU antibody, and substrate turnover was measured at 405 nm on an ELISA reader.

### siRNA

On-TARGETplus siRNA pools specific for EGFR and a nontargeting pool (control) from DharmaFECT were used (Dharmacon, Lafayette, CO, US). Kyse30 cells were transfected with 50 nM final siRNA concentration using Dharmafect reagent 1 for 24 hr. Media were then changed to normal medium without siRNA and Dharmafect, and cells were subjected to further assays.

### IHC and immunofluorescence staining

EpEX- (VU1D9, Cell Signaling Technology, NEB, Frankfurt, Germany, #2929, 1:100), EGFR- (Dianova, Hamburg, Germany, #DLN-08892, 1:200), pERK1/2^Thr202/Tyr204^- (Cell signaling technology; #4370; 1:200), and pAKT^Ser473^-specific antibodies (Cell Signaling technology; #4060; 1:400) were used for IHC and immunofluorescence staining in combination with the avidin-biotin-peroxidase method (Vectastain, Vector laboratories, Burlingame, CA, US) or Alexa Fluor-488- and Alexa Fluor-594-conjugated secondary antibodies. Confocal microscopy images were recorded with a TCS-SP5 system (Leica Microsystems; Wetzlar, Germany). IHC intensity scores were calculated as described [28].

### Flow cytometry, immunoblotting, immunoprecipitation

EpCAM and EGFR were stained with EpCAM- (CD326; BD Biosciences; Heidelberg, Germany, 1:50 dilution in PBS-3% FCS) or EGFR-specific antibodies (Dianova, Hamburg, Germany, #DLN-08892, 1:200), 15 min on ice, washed 3 times in PBS-3% FCS, and stained with FITC-conjugated secondary antibody (Vector Laboratories/Biozol, Eching, Germany; FI-4001; 1:50). Measurement of cell surface expression was performed in a FACSCalibur (BD Pharmingen, Heidelberg, Germany).

Immunoblotting of EpCAM (DAKO/Biozol; Eching, Germany; #M7239; 1:5,000), EGFR (Cell signaling technology; #2232s; 1:1,000), ERK1/2 (Cell signaling technology; #137f5; 1:1,000), pERK1/2^Thr202/Tyr204^, Akt (Cell signaling technology; #9272; 1:1000), pAkt-Ser^473^ (Cell Signaling Technologies; #92725 and #4060), and actin (Santa Cruz, Santa Cruz, CA, US, #sc-47778; 1:5,000) was performed with 10–50 μg of lysate (PBS, 1% triton X-100, Roche complete protease inhibitors) as described [48].

Immunoprecipitation was conducted with precleared cell lysates’ (16,000 rcf, 15′) incubation with EGFR- or EpCAM-specific antibodies (10 μg), EpEX-Fc, or Fc (50 μg) overnight at 4 °C before protein A agarose beads (Thermo scientific Pierce, Munich, Germany, #20333; 100 μl) were added for 2 hr at room temperature. Immunocomplexes were washed 5 times in 25 mM tris, 150 mM NaCl, pH 7.2, boiled in Laemmli sample buffer [117], and loaded on SDS-PAGE.

### Protein expression, purification, and cross-linking

EpEX (aa 24–265) was expressed in Sf9 insect cells (Thermo Scientific) and purified as described [47]. EGFR_ex_ (aa 25–642; gift from Matthew Meyerson; Addgene plasmid #11011) was expressed and purified as reported [118]. For cross-linking, EpEX and EGF (Sigma) (250 pmol) and EGFR_ex_ (50 pmol) were mixed in final volumes of 9 μl of 20 mM HEPES pH 8.0, 100 mM NaCl for 1 hr at 37 °C at 1,000 RPM on a thermomixer. Afterwards, 3.6 μg of BS3 cross-linker (Sigma) was added for 30 min at 37 °C at 1,000 RPM on a thermomixer. Reaction was stopped by adding 1 μl of 1 M Tris pH 8.0 and an additional incubation of 15 min.

### Migration assay, cell proliferation, BrdU incorporation

Migration was assessed as described [54]. Quantification of scratches was performed using ImageJ and MRI wound healing tool. Relative migration was adjusted for proliferation rates. For cell proliferation, 1 × 10^5^ cells were plated in 12-well plates before treatment. At indicated time points, cells were counted in an EVE automatic cell counter (NanoEntek, VWR, Munich, Germany). Measurement of BrdU incorporation was performed as follows.

### Reverse transcriptase PCR analysis

Total RNA was extracted using RNeasy Mini kit coupled with RNase-free DNase set (Qiagen) and reverse transcribed with Reverse transcription kit (Qiagen). The resulting cDNAs were used for PCR using SYBR-Green Master PCR mix in triplicates. PCR and data collection were performed on LightCycler480 (Roche). All quantifications were normalized to an endogenous control GAPDH. The relative quantitation value for each target gene compared to the calibrator for that target is expressed as 2^-(Ct-Cc)^ (Ct and Cc are the mean threshold cycle differences after normalizing to GAPDH).

### Primers used to amplify the genes

E-cadherin-FW 5′-TGC CCA GAA AAT GAA AAA GG-3′

E-cadherin-BW 5′-GTG TAT GTG GCA ATG CGT TC-3′

N-cadherin-FW 5′-GAC AAT GCC CCT CAA GTG TT-3′

N-cadherin-BW 5′-CCA TTA AGC CGA GTG ATG GT-3′

Vimentin-FW 5′-GAG AAC TTT GCC GTT GAA GC-3′

Vimentin-BW 5′-GCT TCC TGT AGG TGG CAA TC-3′

Snail-FW 5′-GCG AGC TGC AGG ACT CTA AT-3′

Snail-BW 5′-CCT CAT CTG ACA GGG AGG TC-3′

Slug-FW 5′-TGA TGA AGA GGA AAG ACT ACAG-3′

Slug-BW 5′-GCT CAC ATA TTC CTT GTC ACA G-3′

Zeb1-FW 5′-TGC ACT GAG TGT GGA AAA GC-3′

Zeb1-BW 5′-TGG TGA TGC TGA AAG AGA CG-3′

Twist-FW 5′-ACA AGC TGA GCA AGA TTC AGA CC-3′

Twist-BW 5′-TCC AGA CCG AGA AGG CGT AG-3′

GAPDH-FW 5′-AGG TCG GAG TCA ACG GAT TT-3′

GAPDH-BW 5′-TAG TTG AGG TCA ATG AAG GG-3′

### Statistical analyses

Results represent means with standard deviations. Significance of differences of two groups was calculated with Student *t* tests in Excel. Significance of differences between more than two groups was calculated with one-way or two-way ANOVA tests and multiple comparisons including Bonferroni or Tukey correction in GraphPad Prism.

### Survival analysis

OS and DFS were calculated in months from the date of diagnosis to death due to any cause (OS) or to first observations of any recurrence or death (DFS). In the absence of an event, patients were censored at the date of the last follow-up visit.

Analysis was performed in R (R: A Language and Environment for Statistical Computing, R Foundation for Statistical Computing, 2017; 3.4.0) together with R-survival package (CRAN). For univariate analysis, IHC scores were included into cox-proportional hazard models after stratification into high and low expressers. Hazard ratios, 95% confidence interval ratios, median survival times, and log-rank *p*-values were included in Kaplan-Meier plots.

## Supporting information

S1 Fig HPV status, tumor localization, and survival of oropharyngeal HNSCCs within the LMU cohort. (A, B) HPV status and tumor localization of each subpopulation are depicted in percent. Chi-squared *p*-value for differences across all four quadrants are mentioned underneath localization and HPV status. Individual *p*-values for differences of single quadrants versus the three remaining quadrants are given under each quadrant. Supporting data are compiled in S1 Data. (C) IHC scores of EGFR and EpCAM expression were assessed in *n* = 105 primary oropharyngeal HNSCCs of the LMU cohort. Expression correlation of EGFR and EpCAM is plotted and subdivided according to a cutoff threshold of 150 (0–300). Percentages of patients within subgroups are indicated in each quadrant. (D) OS and DFS were stratified according to all four quadrants defined in C and are represented as Kaplan-Meier survival curves with *p*-values, hazard ratios, and confidence intervals. DFS, disease-free survival; EGFR, epidermal growth factor receptor; EpCAM, epithelial cell adhesion molecule; HPV, human papillomavirus; HNSCC, head and neck squamous cell carcinoma; IHC, immunohistochemistry; LMU, Ludwig-Maximilians-University; OS, overall survival.
(TIF)

S2 Fig EGFR and EpCAM coexpression in HNSCC cell lines. (A) Cell surface expression of EGFR and EpCAM was assessed by immunostaining and flow cytometry on FaDu, Kyse30, and Cal27 (HNSCC/esophageal) and HCT8 (colon carcinoma) cell lines. Representative histograms of EGFR and EpCAM expression from *n* = 3 independent experiments are shown. Supporting data are compiled in S1 Data. Gating strategy and histogram generation are exemplified in S2 Data. (B) Colocalization of EGFR and EpCAM was assessed by double fluorescent immunostaining of FaDu and Cal27 cells. EGFR: green, EpCAM: red, nucleus: blue (DAPI). Shown are representative pictures in low (left) and high (right) magnifications from *n* = 3 independent experiments. EGFR, epidermal growth factor receptor; EpCAM, epithelial cell adhesion molecule; HNSCC, head and neck squamous cell carcinoma.
(TIF)

S3 Fig EGF treatment of various carcinoma cell lines does not induce EpCAM cleavage. (A) Immunoprecipitation of EpEX from supernatants of Kyse30 and HCT8 cells expressing EpCAM-YFP with or without EGF 1.8 nM for 24 hr. Shown are representative results from *n* = 3 independent experiments. (B) Visualization of CTF-EpCAM-YFP in membrane isolates of Kyse30 and HCT8 cells expressing EpCAM-YFP with or without EGF 1.8 nM for 24 hr. Shown are representative results from *n* = 3 independent experiments. (C) Visualization of EpCAM-YFP, CTF-YFP, and EpICD-YFP in Kyse30 and HCT8 and Kyse30 cells expressing EpCAM-YFP with or without EGF 1.8 nM for 24 hr. Shown are representative results from *n* = 3 independent experiments. (D) Indicated cell lines were treated with EGF 1.8 nM for 24 hr, and cell surface expression of EpCAM was assessed by fluorescence immunostaining and laser scanning confocal microscopy. EpCAM: green, nuclei: blue (DAPI). Shown are representative results from *n* = 3 independent experiments with multiple areas analyzed. EGF, epidermal growth factor; EpCAM, epithelial cellular adhesion molecule; EpCAM-YFP, fusion of EpCAM with yellow fluorescent protein; EpEX, extracellular domain of EpCAM.
(TIF)

S4 Fig EGF treatment of various carcinoma cell lines does not reduce expression of EpCAM. (A-C) Indicated cell lines were treated with (A) EGF 1.8 nM, 18 nM, (B) 9 nM, or (C) TGFα 1.8 nM for 24 or 72 hr. Shown are representative flow cytometry graphs of EGFR and EpCAM cell surface expression. Supporting data are compiled in S1 Data. Gating strategy and histogram generation are exemplified in S3–S6 Figs. (D-E) Indicated cell lines were treated with (D) EGF 1.8 nM or 18 nM for 24 hr or (E) EGF 9 nM for 72 hr. Shown are representative immunoblot results of EpCAM expression. Actin levels served as loading controls. EpCAM expression levels normalized for actin and standardized to control are indicated below immunoblots. EGF, epidermal growth factor; EGFR, EGF receptor; EpCAM, epithelial cell adhesion molecule; TGFα, transforming growth factor alpha.
(TIF)

S5 Fig EpCAM is dispensable for EGF-induced EMT. (A) Wild-type, EpCAM-YFP transfectant, EpCAM knockdown, and control clones of Kyse30 cells were subjected to immunoblotting for EpCAM. Shown is one representative result from *n* = 3 independent experiments. Actin served as loading control. (B) Wild-type, EpCAM-YFP transfectant, EpCAM knockdown, and controls clones of Kyse30 cells were treated with 1.8 nM or 9 nM EGF. Cell morphology was monitored after 48 hr. Shown are representative pictures from *n* = 3 independent experiments. EGF, epidermal growth factor; EMT, epithelial-mesenchymal transition; EpCAM, epithelial cell adhesion molecule; EpCAM-YFP, fusion of EpCAM with yellow fluorescent protein.
(TIF)

S6 Fig Generation and quality control of EpEX-Fc. (A) A fusion consisting of EpEX and the constant region of IgG1 was expressed in HEK293 cells. Cell supernatants were harvested, and EpEX-Fc was purified using protein A agarose beads. (B) Coomassie gel showing EpEX-Fc purity. (C) EpEX-Fc is composed of EpEX and Fc, as determined in immunoblot experiments with the indicated protein concentrations and specific antibodies. (D) EpEX-Fc oligomerizes to form dimers and trimers, as determined in native versus reducing immunoblot experiments with specific antibodies. (E) EpEX-Fc is glycosylated, as determined in immunoblot experiments of cells treated with glycosidase (PNGAse). As a control, HEK293 expressing full-length EpCAM, control HEK293, HCT8, and FaDu cells were similarly treated. EpCAM, epithelial cell adhesion molecule; EpEX, extracellular domain of EpCAM; Fc, fragment crystallizable region; HEK293, human embryonic kidney 293; IgG1, immunoglobulin G1; PNGase, peptide:N-glycanase.
(TIF)

S7 Fig EMT nonresponsive Cal27 cells; EMT marker expression following EGF treatment. (A) Cal27 cells were either kept untreated (control) or were treated with Fc (10 nM), EpEX-Fc, EGF, or combinations with the indicated concentrations. Shown are representative micrograph pictures of cells after 48 hr (Kyse30) and 72 hr (FaDu, Cal27) from *n* = 3 independent experiments. Supporting data are compiled in S1 Data. (C) Cal27 cells were treated with control media and high (9 nM) dose of EGF. Expression of E-cadherin was assessed by immunoblotting after 72 hr. Shown are mean values with SDs from *n* = 3 independent experiments. (C) FaDu and Kyse30 cells were treated with Fc (10 nM), EpEX-Fc, EGF, or combinations with the indicated concentrations. After 6 and 72 hr of treatment, mRNA levels of the indicated transcripts were assessed by qRT-PCR with GAPDH as a housekeeping gene. mRNA levels are represented as relative levels compared to control-treated cells. Shown are mean with SD of *n* = 3 independent experiments performed in triplicate. Supporting data are compiled in S1 Data. EGF, epidermal growth factor; EMT, epithelial-mesenchymal transition; Fc, fragment crystallizable region; NS, not significant; qRT-PCR, quantitative real-time PCR; SD, standard deviation.
(TIF)

S1 Table Clinical parameters of the HNSCC LMU cohort (*n* = 180) including gender, age, p16 expression, TNM stage, smoking habits, and tumor sublocalization. Shown are absolute numbers and percentages referring to the entire cohort. HNSCC, head and neck squamous cell carcinoma; LMU, Ludwig-Maximilians-University; n.d., not determined; TNM, tumor, node, metastasis.
(DOCX)

S1 Data Numerical data for statistical analysis. All numerical data supporting the statistical analyses performed throughout the manuscript were compiled in separated, annotated sheets within one Excel file.
(XLSX)

S2 Data FACS analysis gating strategy and analysis for S2 Fig. FACS gating strategy for the expression of EGFR and EpCAM is displayed, with FSC and SSC, gating of PI-negative cells (FSC and PI) to generate histogram plots for control (“iso”) and antigen expression as shown in S2 Fig. Shown are examples of gating and histograms for FaDu, Kyse30, Cal27, HCT8 cell lines. EGFR, epidermal growth factor receptor; EpCAM, epithelial cell adhesion molecule; FACS, fluorescence-activated cell sorting; FSC, forward scatter; PI, proprium iodide; SSC, side scatter.
(TIF)

S3 Data FACS analysis gating strategy and analysis for S4A Fig. FACS gating strategy for the expression of EpCAM is displayed with FSC and SSC, gating of PI-negative cells (FSC and PI) to generate histogram plots for control (“iso”), and expression of EpCAM as shown in S4A Fig. Shown are examples of gating and histograms for all the indicated cell lines and treatments. EpCAM, epithelial cell adhesion molecule; FACS, fluorescence-activated cell sorting; FSC, forward scatter; PI, proprium iodide; SSC, side scatter.
(TIF)

S4 Data FACS analysis gating strategy and analysis for S4A Fig. FACS gating strategy for the expression of EGFR is displayed with FSC and SSC, gating of PI-negative cells (FSC and PI) to generate histogram plots for control (“iso”) and expression of EpCAM as shown in S4A Fig. Shown are examples of gating and histograms for all the indicated cell lines and treatments. EGFR, epidermal growth factor receptor; EpCAM, epithelial cell adhesion molecule; FACS, fluorescence-activated cell sorting; FSC, forward scatter; PI, proprium iodide; SSC, side scatter.
(TIF)

S5 Data FACS analysis gating strategy and analysis for S4B Fig. FACS gating strategy for the expression of EpCAM is displayed with FSC and SSC, gating of PI-negative cells (FSC and PI) to generate histogram plots for control (“iso”), and expression of EpCAM as shown in S4B Fig. Shown are examples of gating and histograms for all the indicated cell lines. EpCAM, epithelial cell adhesion molecule; FACS, fluorescence-activated cell sorting; FSC, forward scatter; PI, proprium iodide; SSC, side scatter.
(TIF)

S6 Data FACS analysis gating strategy and analysis for S4C Fig. FACS gating strategy for the expression of EpCAM is displayed with FSC and SSC, gating of PI-negative cells (FSC and PI) to generate histogram plots for control (“iso”), and expression of EpCAM as shown in S4C Fig. Shown are examples of gating and histograms for all the indicated cell lines. EpCAM, epithelial cell adhesion molecule; FACS, fluorescence-activated cell sorting; FSC, forward scatter; PI, proprium iodide; SSC, side scatter.
(TIF)

## Abbreviations

AJCC: American Joint Committee on Cancer
BrdU: bromodeoxyuridine
CRISPR-Cas9: clustered regularly interspaced short palindromic repeat/CRISPR-associated 9
CSC: cancer stem cell
CTF: C-terminal fragment
DAPT: N-[N-(3,5-Difluorophenacetyl)-L-alanyl]-S-phenylglycine t-butyl ester
DFS: disease-free survival
EGF: epidermal growth factor
EGFR: EGF receptor
EGFR_ex_: extracellular domain of EGFR
EMT: epithelial-mesenchymal transition
EMT-TF: EMT transcription factors
EpCAM K.O.1: *EPCAM*-knockout clone 1
EpCAM: epithelial cell adhesion molecule
EpCAM-YFP: fusion of EpCAM with yellow fluorescence protein
EpEX: extracellular domain of EpCAM
EpICD: intracellular domain of EpCAM
ERK1/2: extracellular signal–regulated kinase 1/2
Fc: fragment crystallizable region
FDA: Food and Drug Administration
GFP: green fluorescent protein
HC: heavy chain
HCT8WT: HCT8 wild type
HEK293: human embryonic kidney 293
HNSCC: head and neck squamous cell carcinoma
HPV: human papillomavirus
IHC: immunohistochemistry
Lef-1: lymphoid enhancer-binding factor 1
LMU: Ludwig-Maximilians-University
MEK: MAPK–ERK kinase
MW: molecular mass
OS: overall survival
pAKT: phosphorylated AKT
pEMT: partial EMT
pERK1/2: phosphorylated extracellular signal–regulated kinase 1/2
PI3: phosphoinositide-3
qRT-PCR: quantitative real-time PCR
Raf: rapidly accelerated fibrosarcoma kinase
Ras: rat sarcoma gene
RIP: regulated intramembrane proteolysis
shRNA: short hairpin RNA
siRNA: small interfering RNA
TCGA: The Cancer Genome Atlas
TGFα: transforming growth factor alpha
TKI: tyrosine kinase inhibitor
TNM: tumor, node, metastasis
y: year
YFP: yellow fluorescence protein
Zeb1: zinc finger E-box-binding homeobox 1
