# Supplementary figures and images for: EpCAM ectodomain EpEX is a ligand of EGFR that counteracts EGF-mediated epithelial-mesenchymal transition through modulation of phospho-ERK1/2 in head and neck cancers
Source: PLoS Biol. 2018 Sep 27;16(9):e2006624. doi: 10.1371/journal.pbio.2006624 (PMC6177200; doi:10.1371/journal.pbio.2006624)

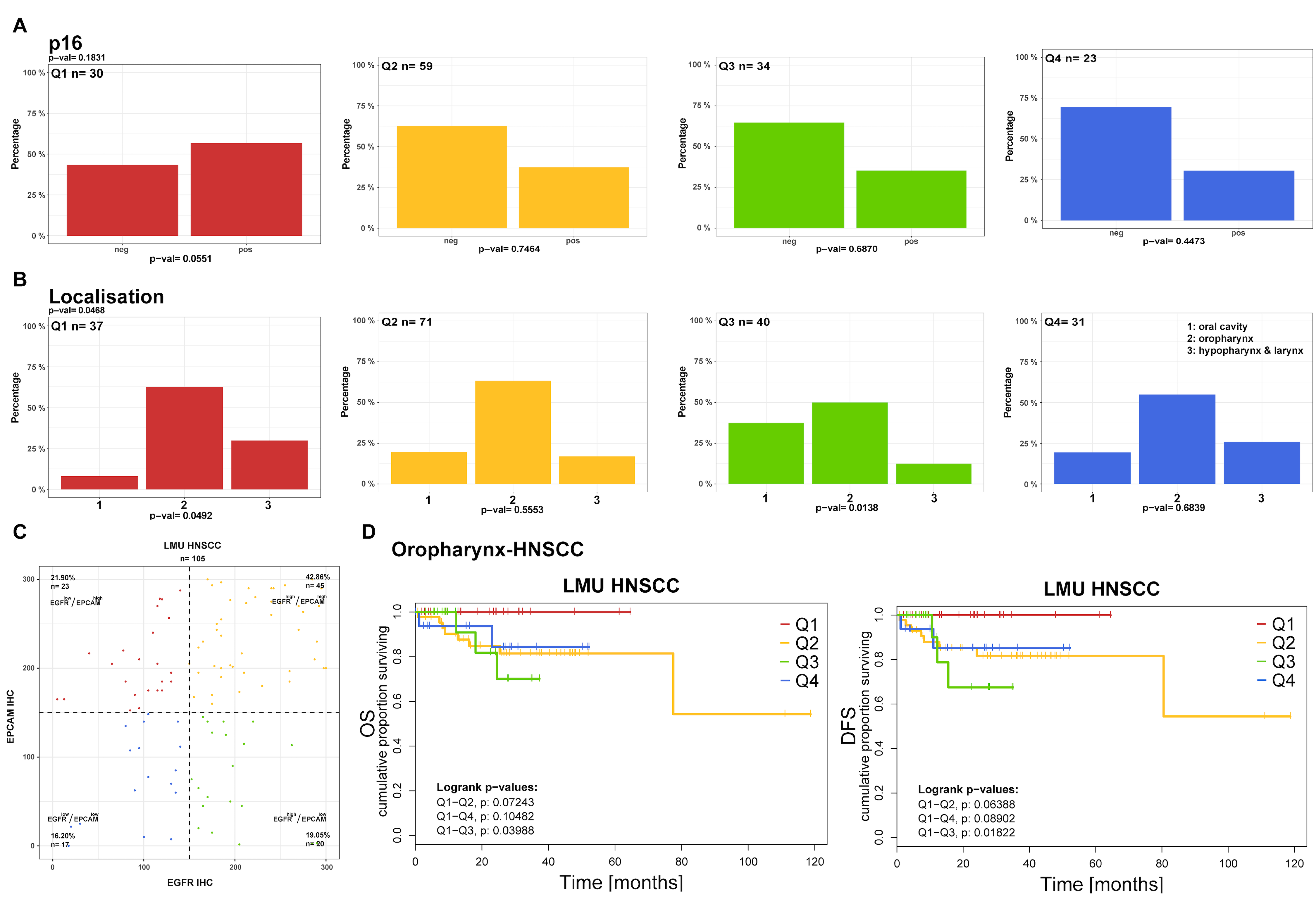

Supplement: S1 Fig — (A, B) HPV status and tumor localization of each subpopulation are depicted in percent. Chi-squared p-value for differences across all four quadrants are mentioned underneath localization and HPV status. Individual p-values for differences of single quadrants versus the three remaining quadrants are given under each quadrant. Supporting data are compiled in S1 Data. (C) IHC scores of EGFR and EpCAM expression were assessed in n = 105 primary oropharyngeal HNSCCs of the LMU cohort. Expression correlation of EGFR and EpCAM is plotted and subdivided according to a cutoff threshold of 150 (0–300). Percentages of patients within subgroups are indicated in each quadrant. (D) OS and DFS were stratified according to all four quadrants defined in C and are represented as Kaplan-Meier survival curves with p-values, hazard ratios, and confidence intervals. DFS, disease-free survival; EGFR, epidermal growth factor receptor; EpCAM, epithelial cell adhesion molecule; HPV, human papillomavirus; HNSCC, head and neck squamous cell carcinoma; IHC, immunohistochemistry; LMU, Ludwig-Maximilians-University; OS, overall survival. (TIF) [file pbio.2006624.s001.tif]

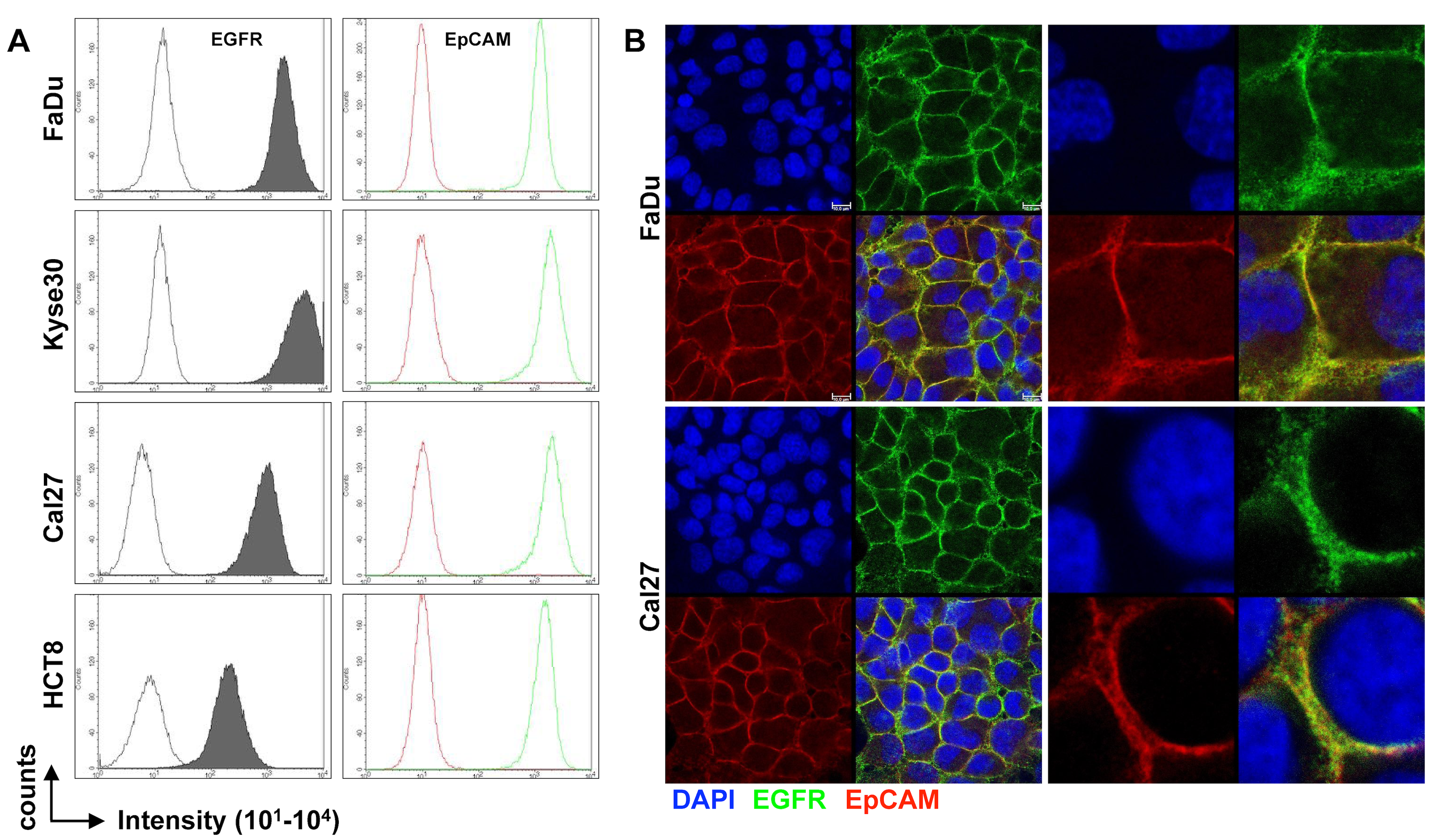

Supplement: S2 Fig — (A) Cell surface expression of EGFR and EpCAM was assessed by immunostaining and flow cytometry on FaDu, Kyse30, and Cal27 (HNSCC/esophageal) and HCT8 (colon carcinoma) cell lines. Representative histograms of EGFR and EpCAM expression from n = 3 independent experiments are shown. Supporting data are compiled in S1 Data. Gating strategy and histogram generation are exemplified in S2 Data. (B) Colocalization of EGFR and EpCAM was assessed by double fluorescent immunostaining of FaDu and Cal27 cells. EGFR: green, EpCAM: red, nucleus: blue (DAPI). Shown are representative pictures in low (left) and high (right) magnifications from n = 3 independent experiments. EGFR, epidermal growth factor receptor; EpCAM, epithelial cell adhesion molecule; HNSCC, head and neck squamous cell carcinoma. (TIF) [file pbio.2006624.s002.tif]

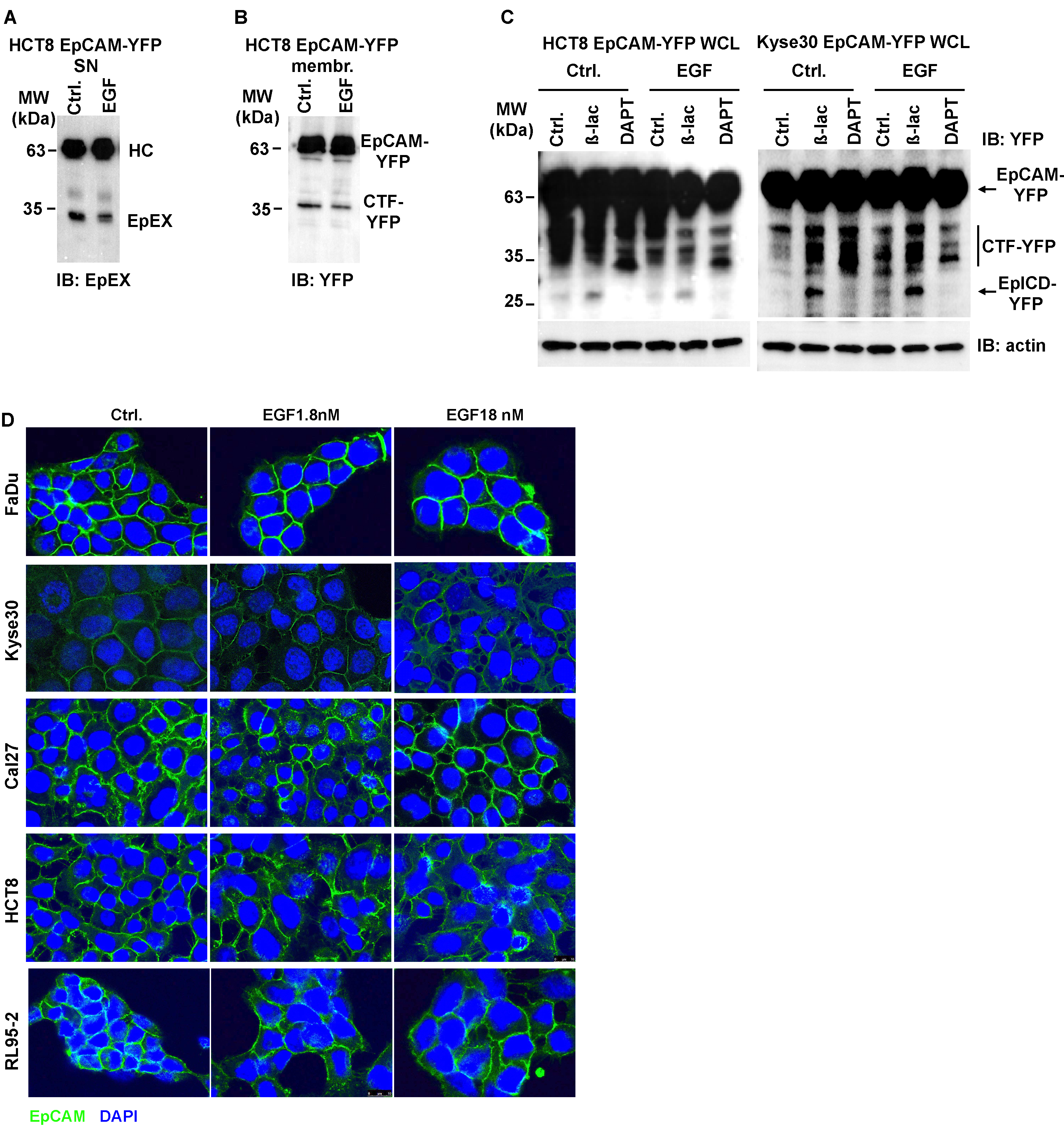

Supplement: S3 Fig — (A) Immunoprecipitation of EpEX from supernatants of Kyse30 and HCT8 cells expressing EpCAM-YFP with or without EGF 1.8 nM for 24 hr. Shown are representative results from n = 3 independent experiments. (B) Visualization of CTF-EpCAM-YFP in membrane isolates of Kyse30 and HCT8 cells expressing EpCAM-YFP with or without EGF 1.8 nM for 24 hr. Shown are representative results from n = 3 independent experiments. (C) Visualization of EpCAM-YFP, CTF-YFP, and EpICD-YFP in Kyse30 and HCT8 and Kyse30 cells expressing EpCAM-YFP with or without EGF 1.8 nM for 24 hr. Shown are representative results from n = 3 independent experiments. (D) Indicated cell lines were treated with EGF 1.8 nM for 24 hr, and cell surface expression of EpCAM was assessed by fluorescence immunostaining and laser scanning confocal microscopy. EpCAM: green, nuclei: blue (DAPI). Shown are representative results from n = 3 independent experiments with multiple areas analyzed. EGF, epidermal growth factor; EpCAM, epithelial cellular adhesion molecule; EpCAM-YFP, fusion of EpCAM with yellow fluorescent protein; EpEX, extracellular domain of EpCAM. (TIF) [file pbio.2006624.s003.tif]

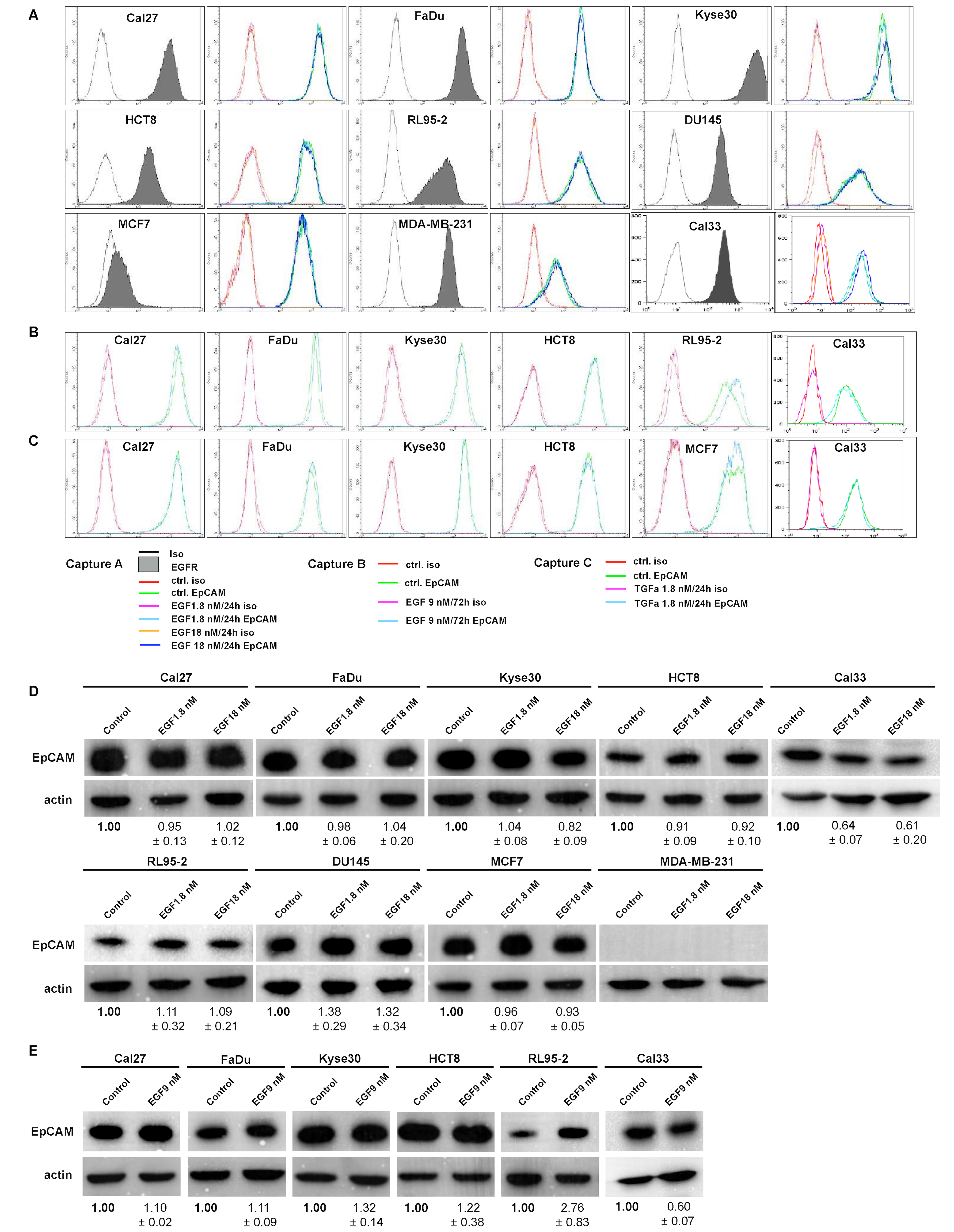

Supplement: S4 Fig — (A-C) Indicated cell lines were treated with (A) EGF 1.8 nM, 18 nM, (B) 9 nM, or (C) TGFα 1.8 nM for 24 or 72 hr. Shown are representative flow cytometry graphs of EGFR and EpCAM cell surface expression. Supporting data are compiled in S1 Data. Gating strategy and histogram generation are exemplified in S3–S6 Figs. (D-E) Indicated cell lines were treated with (D) EGF 1.8 nM or 18 nM for 24 hr or (E) EGF 9 nM for 72 hr. Shown are representative immunoblot results of EpCAM expression. Actin levels served as loading controls. EpCAM expression levels normalized for actin and standardized to control are indicated below immunoblots. EGF, epidermal growth factor; EGFR, EGF receptor; EpCAM, epithelial cell adhesion molecule; TGFα, transforming growth factor alpha. (TIF) [file pbio.2006624.s004.tif]

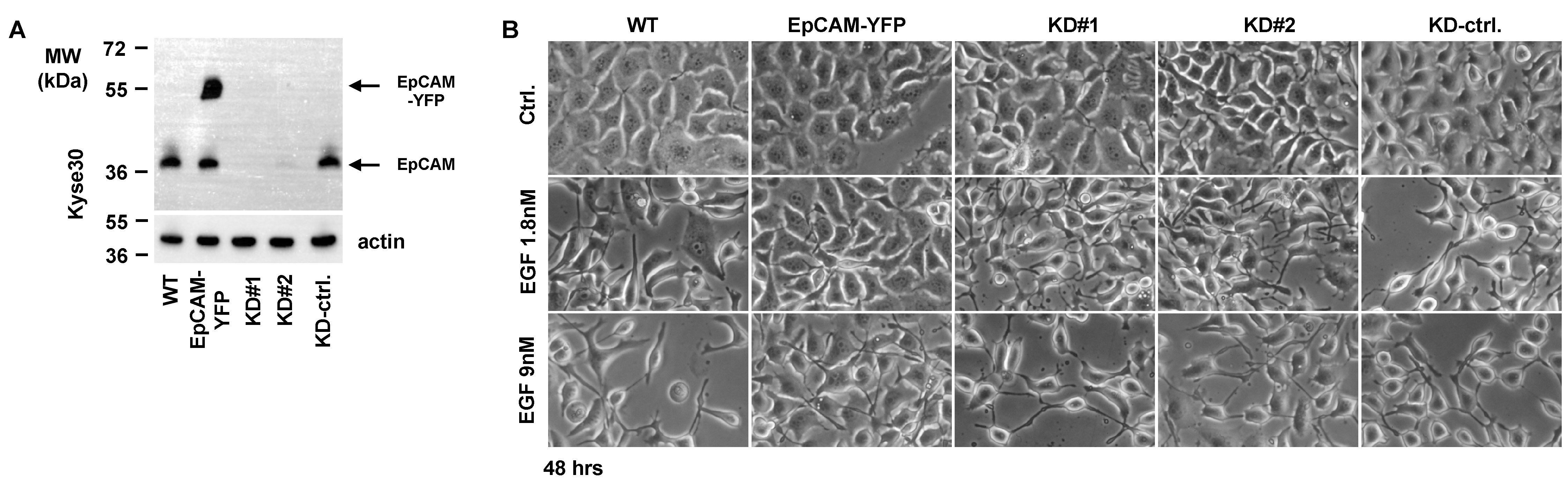

Supplement: S5 Fig — (A) Wild-type, EpCAM-YFP transfectant, EpCAM knockdown, and control clones of Kyse30 cells were subjected to immunoblotting for EpCAM. Shown is one representative result from n = 3 independent experiments. Actin served as loading control. (B) Wild-type, EpCAM-YFP transfectant, EpCAM knockdown, and controls clones of Kyse30 cells were treated with 1.8 nM or 9 nM EGF. Cell morphology was monitored after 48 hr. Shown are representative pictures from n = 3 independent experiments. EGF, epidermal growth factor; EMT, epithelial-mesenchymal transition; EpCAM, epithelial cell adhesion molecule; EpCAM-YFP, fusion of EpCAM with yellow fluorescent protein. (TIF) [file pbio.2006624.s005.tif]

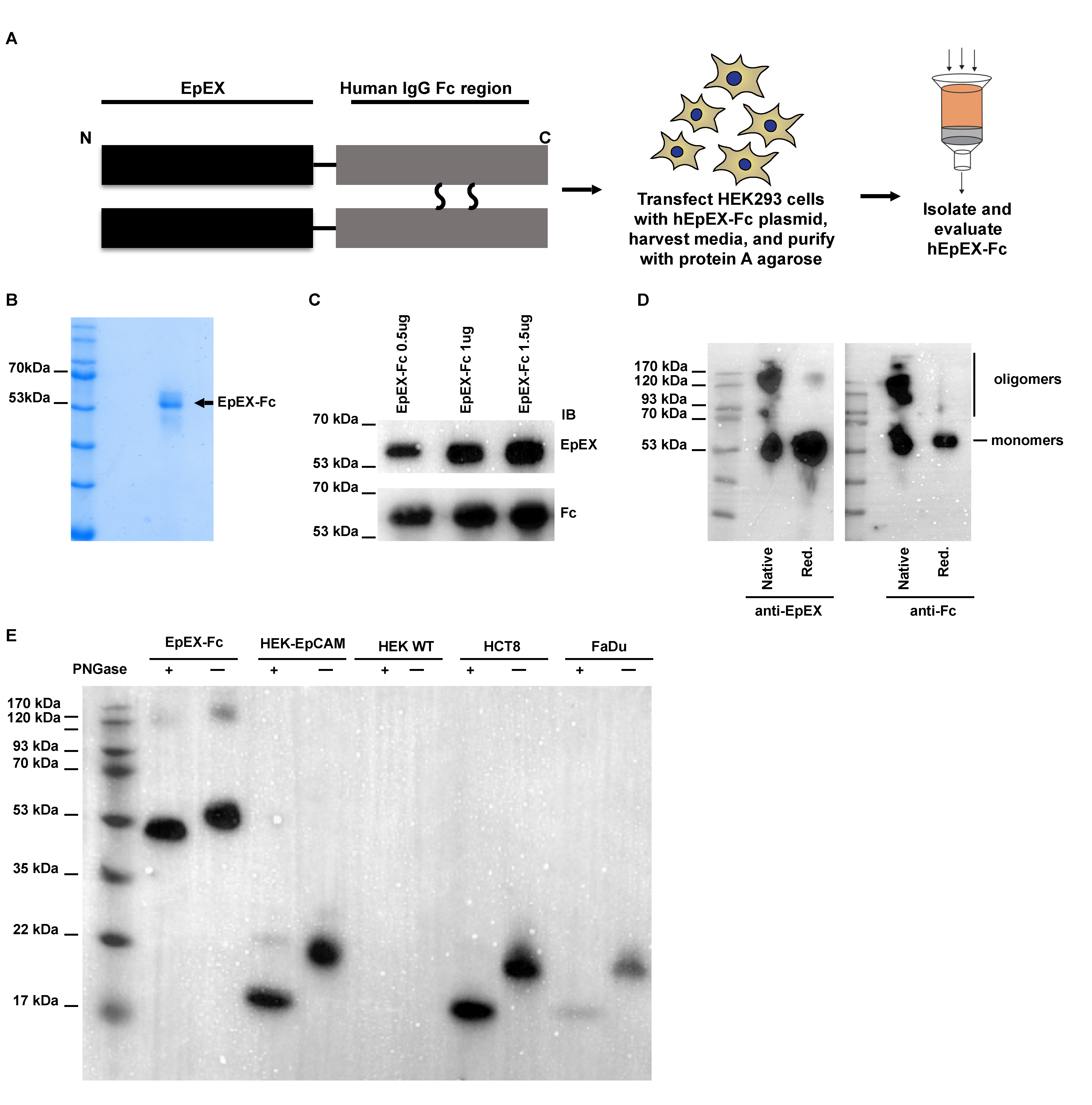

Supplement: S6 Fig — (A) A fusion consisting of EpEX and the constant region of IgG1 was expressed in HEK293 cells. Cell supernatants were harvested, and EpEX-Fc was purified using protein A agarose beads. (B) Coomassie gel showing EpEX-Fc purity. (C) EpEX-Fc is composed of EpEX and Fc, as determined in immunoblot experiments with the indicated protein concentrations and specific antibodies. (D) EpEX-Fc oligomerizes to form dimers and trimers, as determined in native versus reducing immunoblot experiments with specific antibodies. (E) EpEX-Fc is glycosylated, as determined in immunoblot experiments of cells treated with glycosidase (PNGAse). As a control, HEK293 expressing full-length EpCAM, control HEK293, HCT8, and FaDu cells were similarly treated. EpCAM, epithelial cell adhesion molecule; EpEX, extracellular domain of EpCAM; Fc, fragment crystallizable region; HEK293, human embryonic kidney 293; IgG1, immunoglobulin G1; PNGase, peptide:N-glycanase. (TIF) [file pbio.2006624.s006.tif]

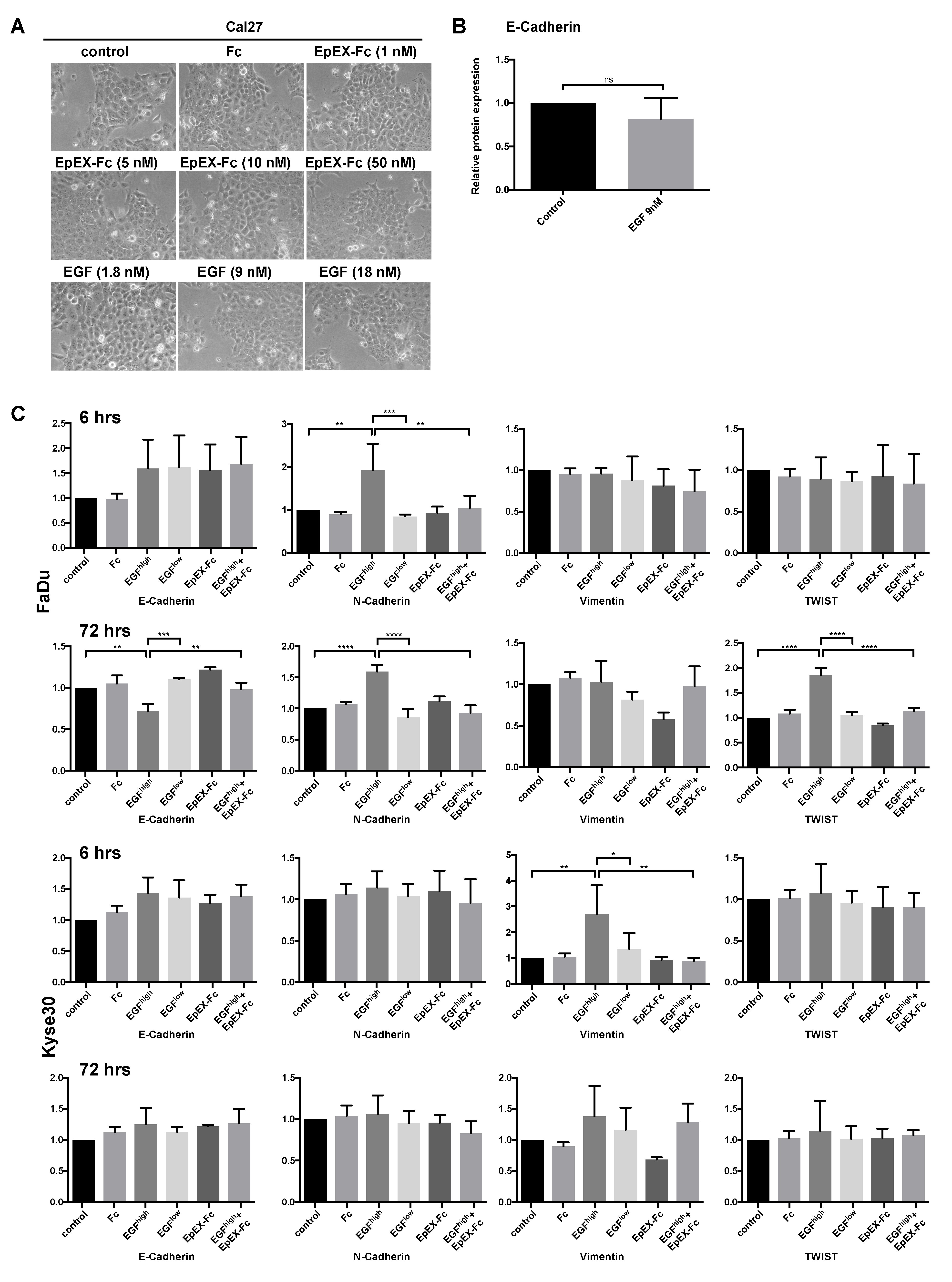

Supplement: S7 Fig — (A) Cal27 cells were either kept untreated (control) or were treated with Fc (10 nM), EpEX-Fc, EGF, or combinations with the indicated concentrations. Shown are representative micrograph pictures of cells after 48 hr (Kyse30) and 72 hr (FaDu, Cal27) from n = 3 independent experiments. Supporting data are compiled in S1 Data. (C) Cal27 cells were treated with control media and high (9 nM) dose of EGF. Expression of E-cadherin was assessed by immunoblotting after 72 hr. Shown are mean values with SDs from n = 3 independent experiments. (C) FaDu and Kyse30 cells were treated with Fc (10 nM), EpEX-Fc, EGF, or combinations with the indicated concentrations. After 6 and 72 hr of treatment, mRNA levels of the indicated transcripts were assessed by qRT-PCR with GAPDH as a housekeeping gene. mRNA levels are represented as relative levels compared to control-treated cells. Shown are mean with SD of n = 3 independent experiments performed in triplicate. Supporting data are compiled in S1 Data. EGF, epidermal growth factor; EMT, epithelial-mesenchymal transition; Fc, fragment crystallizable region; NS, not significant; qRT-PCR, quantitative real-time PCR; SD, standard deviation. (TIF) [file pbio.2006624.s007.tif]

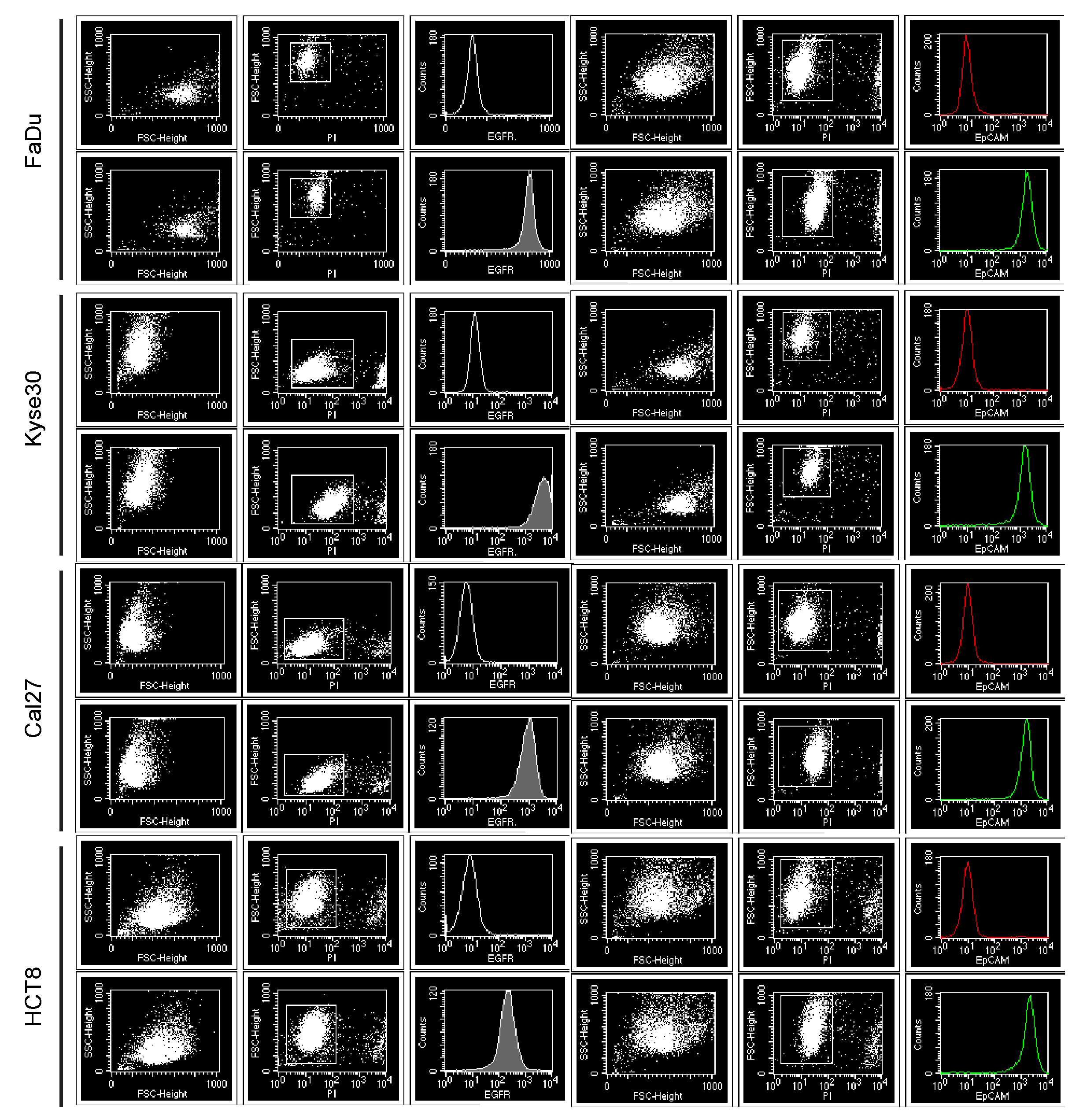

Supplement: S2 Data — FACS gating strategy for the expression of EGFR and EpCAM is displayed, with FSC and SSC, gating of PI-negative cells (FSC and PI) to generate histogram plots for control (“iso”) and antigen expression as shown in S2 Fig. Shown are examples of gating and histograms for FaDu, Kyse30, Cal27, HCT8 cell lines. EGFR, epidermal growth factor receptor; EpCAM, epithelial cell adhesion molecule; FACS, fluorescence-activated cell sorting; FSC, forward scatter; PI, proprium iodide; SSC, side scatter. (TIF) [file pbio.2006624.s010.tif]

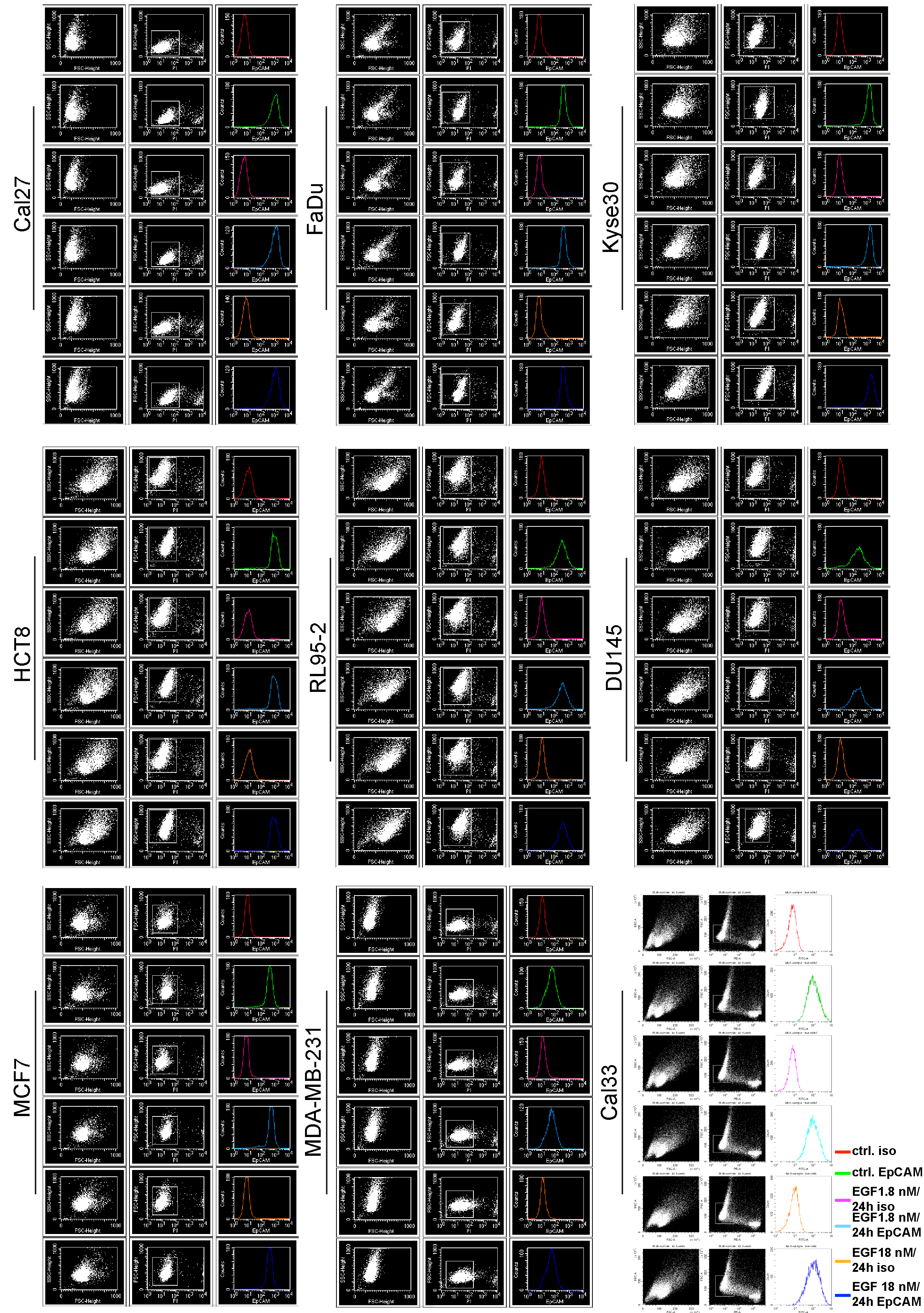

Supplement: S3 Data — FACS gating strategy for the expression of EpCAM is displayed with FSC and SSC, gating of PI-negative cells (FSC and PI) to generate histogram plots for control (“iso”), and expression of EpCAM as shown in S4A Fig. Shown are examples of gating and histograms for all the indicated cell lines and treatments. EpCAM, epithelial cell adhesion molecule; FACS, fluorescence-activated cell sorting; FSC, forward scatter; PI, proprium iodide; SSC, side scatter. (TIF) [file pbio.2006624.s011.tif]

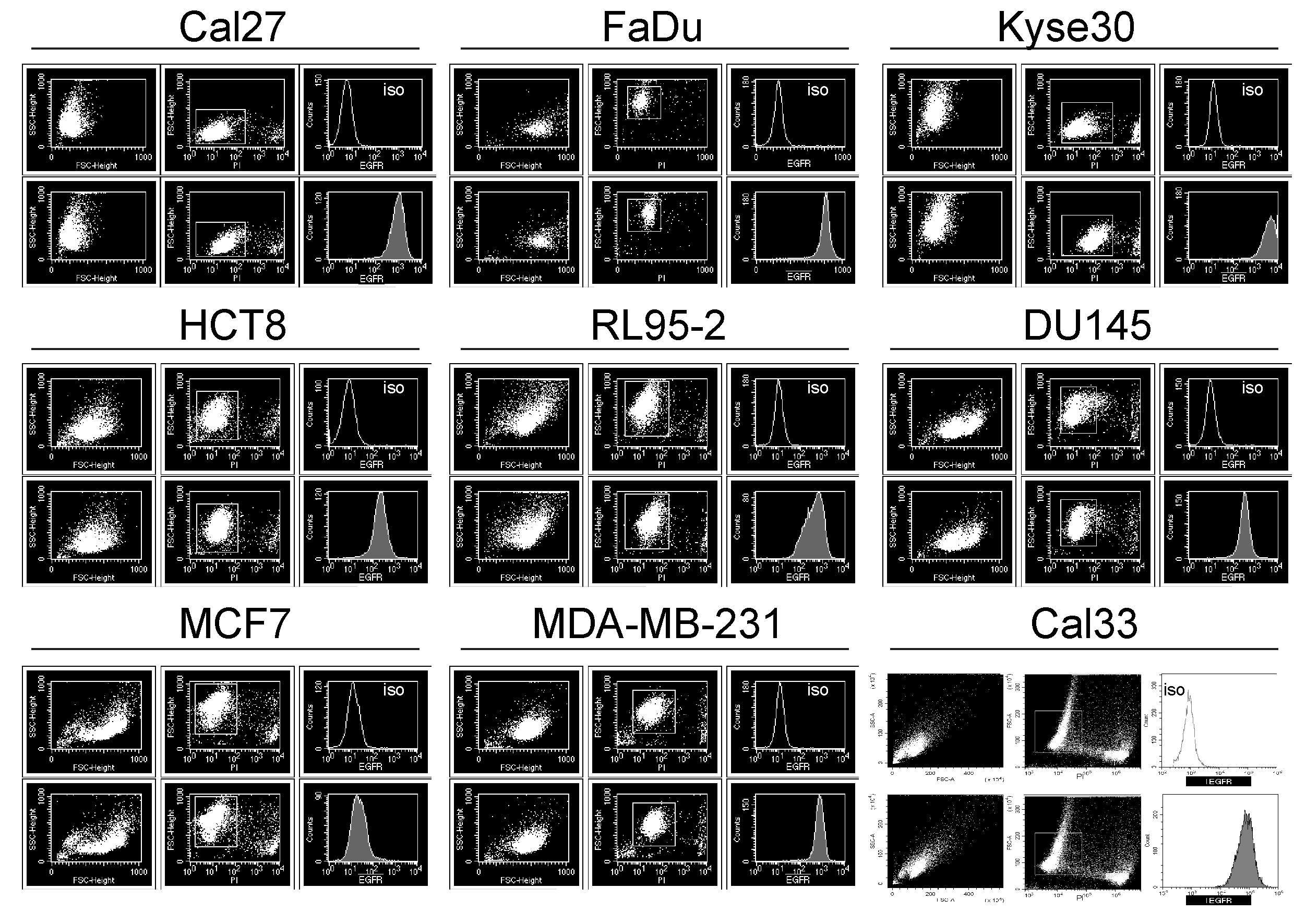

Supplement: S4 Data — FACS gating strategy for the expression of EGFR is displayed with FSC and SSC, gating of PI-negative cells (FSC and PI) to generate histogram plots for control (“iso”) and expression of EpCAM as shown in S4A Fig. Shown are examples of gating and histograms for all the indicated cell lines and treatments. EGFR, epidermal growth factor receptor; EpCAM, epithelial cell adhesion molecule; FACS, fluorescence-activated cell sorting; FSC, forward scatter; PI, proprium iodide; SSC, side scatter. (TIF) [file pbio.2006624.s012.tif]

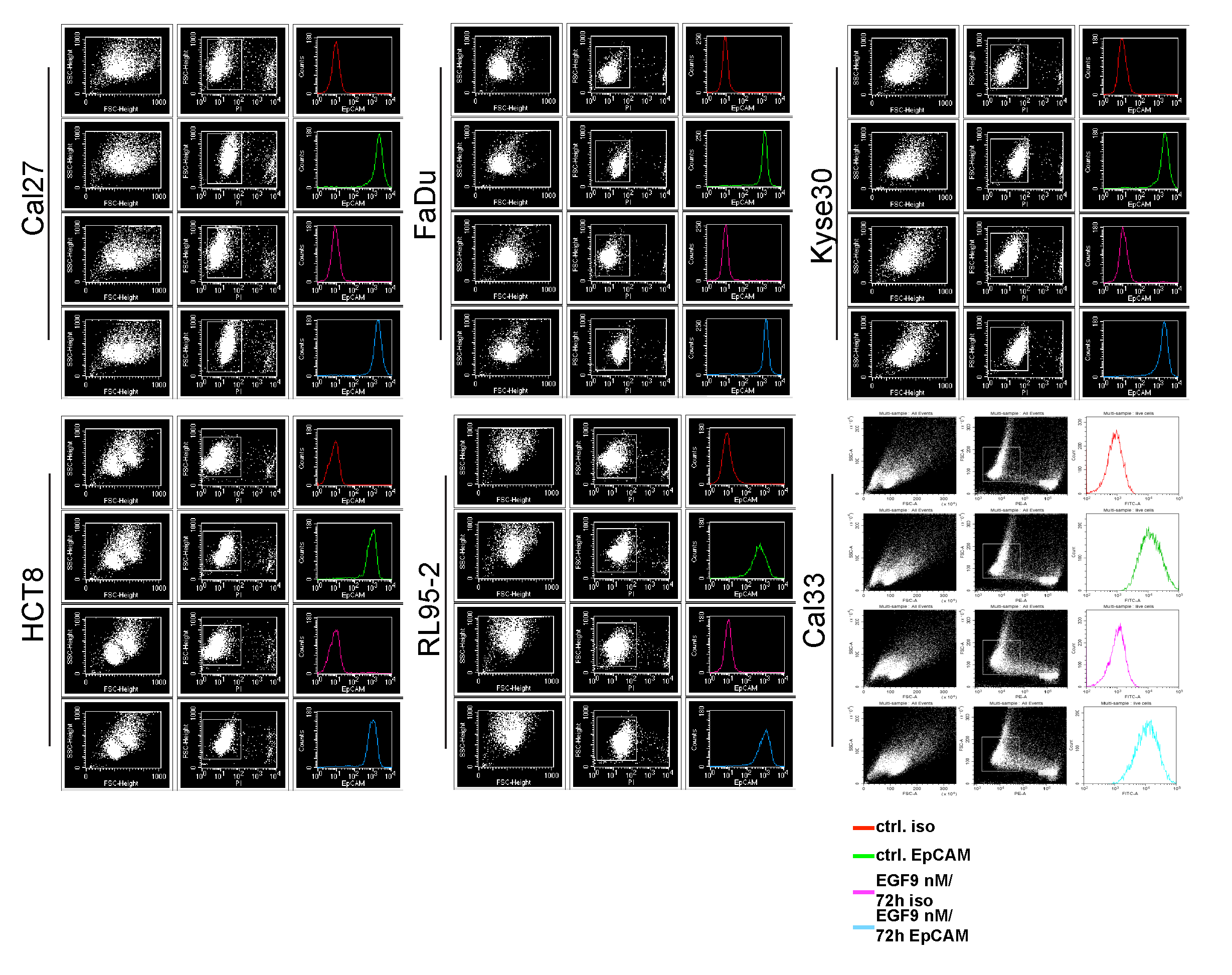

Supplement: S5 Data — FACS gating strategy for the expression of EpCAM is displayed with FSC and SSC, gating of PI-negative cells (FSC and PI) to generate histogram plots for control (“iso”), and expression of EpCAM as shown in S4B Fig. Shown are examples of gating and histograms for all the indicated cell lines. EpCAM, epithelial cell adhesion molecule; FACS, fluorescence-activated cell sorting; FSC, forward scatter; PI, proprium iodide; SSC, side scatter. (TIF) [file pbio.2006624.s013.tif]

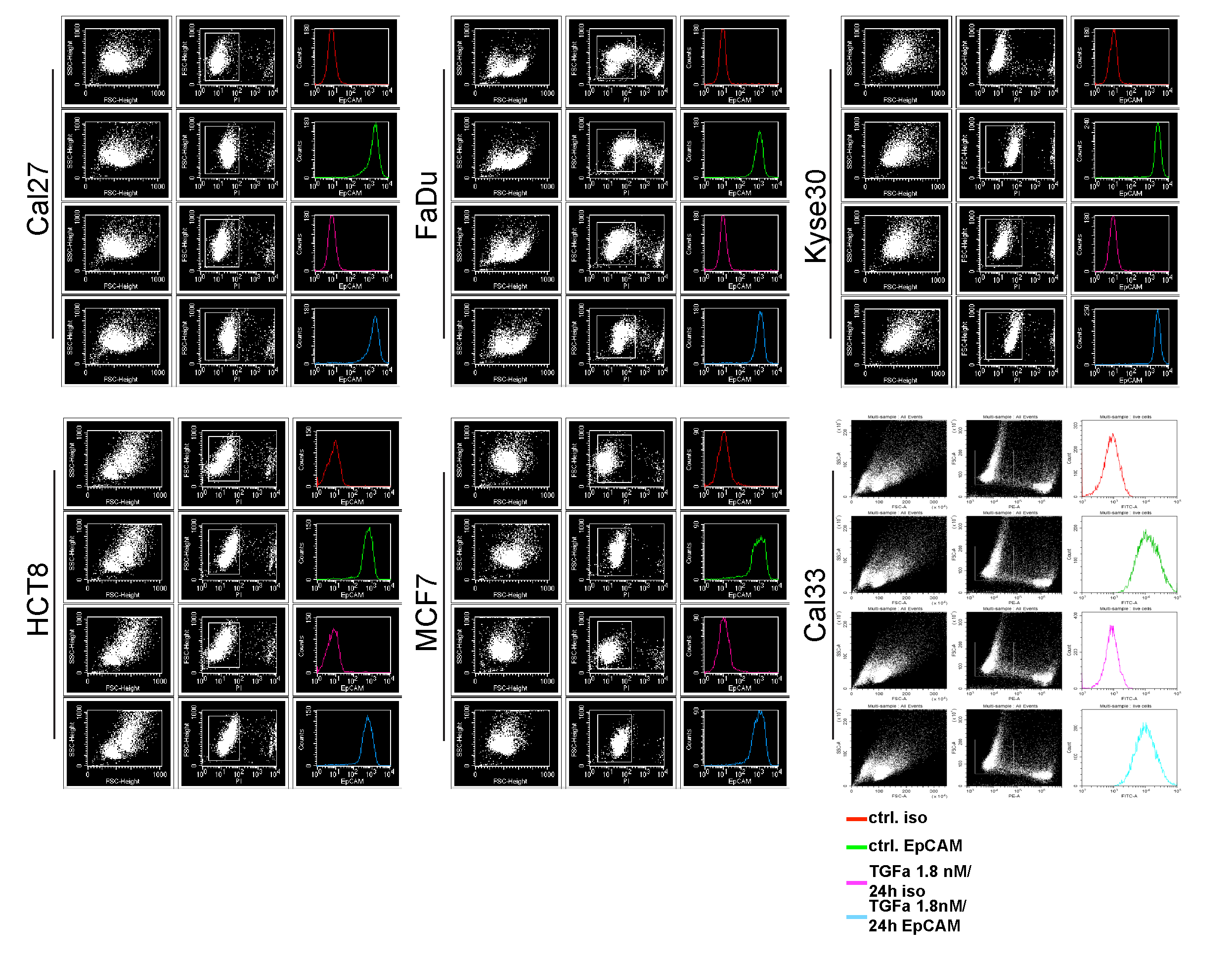

Supplement: S6 Data — FACS gating strategy for the expression of EpCAM is displayed with FSC and SSC, gating of PI-negative cells (FSC and PI) to generate histogram plots for control (“iso”), and expression of EpCAM as shown in S4C Fig. Shown are examples of gating and histograms for all the indicated cell lines. EpCAM, epithelial cell adhesion molecule; FACS, fluorescence-activated cell sorting; FSC, forward scatter; PI, proprium iodide; SSC, side scatter. (TIF) [file pbio.2006624.s014.tif]
