# Supplementary material for: EpCAM ectodomain EpEX is a ligand of EGFR that counteracts EGF-mediated epithelial-mesenchymal transition through modulation of phospho-ERK1/2 in head and neck cancers
Source: PLoS Biol. 2018 Sep 27;16(9):e2006624. doi: 10.1371/journal.pbio.2006624 (PMC6177200; doi:10.1371/journal.pbio.2006624)
Supplement: S1 Table — Shown are absolute numbers and percentages referring to the entire cohort. HNSCC, head and neck squamous cell carcinoma; LMU, Ludwig-Maximilians-University; n.d., not determined; TNM, tumor, node, metastasis. (DOCX) [file pbio.2006624.s008.docx]

**Supplementary Table 1**: Clinical parameters of HNSCC LMU cohort (n=180) including gender, age, p16 expression, TNM stage, smoking habits, and tumor sub-localization. n.d. not determined. Shown are absolute numbers and percentages referring to the entire cohort.

| HNSCC cohort | | | | | |
| --- | --- | --- | --- | --- | --- |
| Gender | **Male** | **Female** | | |  |
| %  absolute | 78.9  142 | 21.1  38 | | |  |
| Age | **<50** | **50-69** | **≥70** | |  |
| %  absolute | 14.3  25 | 63.4  116 | 22.3  39 | |  |
| P16 | **Negative** | **Positive** | **n.p.** | |  |
| %  absolute | 48.9  88 | 32.2  58 | 18.9  34 | |  |
| T-Stage | **pT1-2** | **pT3-4** | **pTx** | |  |
| %  absolute | 50.9  92 | 48.0  86 | 1.1  2 | |  |
| N-Stage | **N0** | **N+** | **Nx** | |  |
| %  absolute | 39.7  71 | 59.7  107 | 0.6  12 | |  |
| M-Stage | **cM0** | **cM+** | **cMx** | |  |
| %  absolute | 96.1  173 | 2.2  4 | 1.7  3 | |  |
| Smoking Status | **Never** | **Former** | **Current** | | **n.d.** |
| %  absolute | 11.1  20 | 57.8  104 | 25.0  45 | | 6.1  11 |
| Localization | **Oral Cavity** | **Oropharynx** | **Hypopharynx**  **& Larynx** | |  |
| %  absolute | 21.2  38 | 58.8  106 | | 20.0  36 | |
